# KSHV promotes leukocyte adhesion and transendothelial migration via induction of VCAM1

**DOI:** 10.1128/mbio.01499-26

**Published:** 2026-09-08

**Authors:** Zhigang Zhang, Kurtis M. Host, Jason Wong, Penny Anders, Blossom Damania

**Affiliations:** 1 Lineberger Comprehensive Cancer Center and Department of Microbiology & Immunology, University of North Carolina at Chapel Hill 318275, Chapel Hill, North Carolina, USA; Duke University School of Medicine, Durham, North Carolina, USA

**Keywords:** Kaposi's sarcoma-associated herpesvirus, Kaposi sarcoma, VCAM1, NF-κB, transendothelial migration, leukocytes

## Abstract

Kaposi’s sarcoma-associated herpesvirus (KSHV) and Epstein-Barr virus are gammaherpesviruses associated with multiple human cancers. Kaposi’s sarcoma (KS) is a human malignancy associated with KSHV infection. KS lesions are characterized by proliferating endothelial-derived spindle cells, leaky blood vessels, and inflammatory infiltrating leukocytes. The inflammatory leukocytes secrete cytokines and growth factors that may contribute to the survival and proliferation of tumor cells. Unless exposed to inflammatory cytokines, resting endothelial cells usually do not express adhesion molecules. Here, we identify a novel mechanistic link demonstrating that KSHV-infected latent endothelial cells express vascular cell adhesion molecule (VCAM1) and induce leukocyte adhesion and transendothelial migration (TEM) in a VCAM1-dependent manner. Inhibition of VCAM1 function using siRNA knockdown or antibody blockade impairs leukocyte adhesion and TEM, suggesting that cell adhesion and TEM are mediated by VCAM1. VCAM1 expression in KSHV-infected endothelial cells involves the non-canonical NF-кB (NF-κB2) pathway because depletion of NIK, IKKα, RelB, or NF-κB p52 via siRNA results in a decrease of VCAM1 levels, as well as a corresponding impairment of cell adhesion and TEM. Thus, KSHV-infected endothelial cells likely promote the infiltration of immune cells by modulating the expression of adhesion factors like VCAM1 on their surface. Moreover, we report that KSHV vFLIP, a latency protein encoded by the virus, directly induces VCAM1 expression and promotes leukocyte adhesion and transendothelial migration. We further demonstrate that endothelial cells infected with a vFLIP-deleted virus had reduced VCAM1 expression and reduced leukocyte adhesion and transendothelial migration.

**IMPORTANCE:**

Kaposi’s sarcoma-associated herpesvirus (KSHV) is associated with the development of Kaposi’s sarcoma (KS). KS tumors are characterized by proliferating endothelial-derived spindle cells and infiltrating leukocytes, which secrete cytokines and growth factors that may contribute to the proliferation of tumor cells. Unless exposed to inflammatory cytokines, resting endothelial cells usually do not express adhesion molecules. Here, we demonstrate that KSHV-infected latent endothelial cells induce the expression of vascular cell adhesion molecule, which promotes leukocyte adhesion and transendothelial migration.

## INTRODUCTION

Kaposi’s sarcoma (KS) is a tumor of endothelial cell (EC) origin most commonly arising in immunocompromised individuals. Kaposi’s sarcoma-associated herpesvirus (KSHV) and Epstein-Barr virus (EBV) are double-stranded DNA gammaherpesviruses. KSHV was identified as the etiologic agent of all forms of KS (classic KS, endemic KS, iatrogenic/transplant KS, and epidemic/AIDS-KS) (1, 2). Although the KS lesion is typically viewed on the skin, KS can also impact the mucosa and internal organs (3).

Like other herpesviruses, KSHV exhibits both a latent and lytic phase. The KS lesion is composed of spindle-shaped ECs and a profusion of neovascular spaces that are weak, allowing leakage of fluids into the extravascular spaces (4–6). In addition to spindle cells and neovascular spaces, the KS lesion is also filled with inflammatory cells, including T, B, and monocytic cells (7–11). Most of the spindle cells in a KS lesion are infected with latent KSHV, with only a small percentage of cells supporting lytic replication (3). Thus, KSHV proteins expressed during latency are major contributors to KS. We have previously shown that KSHV infection induces vascular permeability in endothelial cells (12).

Inflammation has been found to play an integral part in the development and progression of KS. In the early stages of KS, there is extensive inflammatory infiltrate, including an influx of interferon-γ-producing T cells and the production of Th1-inflammatory cytokines (8). Inflammatory cytokines stimulate endothelial cells to produce angiogenic factors, thereby promoting KS progression (13). KS has also been found to localize to surgical sites or areas of skin trauma, further emphasizing the importance of inflammation to KS development (14).

Resting endothelial cells usually do not express adhesion molecules. However, activated endothelial cells express adhesion molecules that can promote leukocytes to undergo transendothelial migration (TEM). TEM involves leukocyte recruitment, adhesion, and transendothelial migration through the endothelium (15, 16). Activated endothelial cells express adhesion molecules such as vascular cell adhesion molecule (VCAM1) (16, 17). VCAM1 belongs to the immunoglobulin (Ig) superfamily of proteins, containing several extracellular Ig-like domains, a transmembrane region, and a cytoplasmic domain (18, 19). VCAM1 expressed on the luminal and lateral sides of endothelial cells binds to the integrin α4β1 found on leukocytes (20). Binding of VCAM1 to α4β1 slows leukocyte rolling and promotes the disassembly of adherence junctions. With increased permeability, leukocytes can migrate through the endothelial cell barrier, thereby accomplishing TEM (21).

Induction of endothelial VCAM1 expression involves the activation of the NF-κB pathway (22). NF-κB transcription factors are activated via canonical and non-canonical pathways. A subset of TNFα receptor superfamily members stimulates the activation of the non-canonical pathway by activating NF-κB-inducing kinase (NIK). NIK phosphorylates and activates IKKα, leading to the selective phosphorylation of the carboxy-terminal serine residues on p100. This phosphorylation event triggers the degradation of p100 and the generation of p52, which leads to the translocation of NF-κB p52 and RelB to the nucleus (23–29).

In this study, we report that latent KSHV infection drives VCAM1 expression via the NF-κB2 pathway, thereby promoting leukocyte adhesion and TEM. Our findings uncover a novel strategy by which KSHV modulates leukocyte trafficking.

## RESULTS

### KSHV infection enhances leukocyte adhesion and promotes TEM in a VCAM1-dependent manner

**Fig 1:**
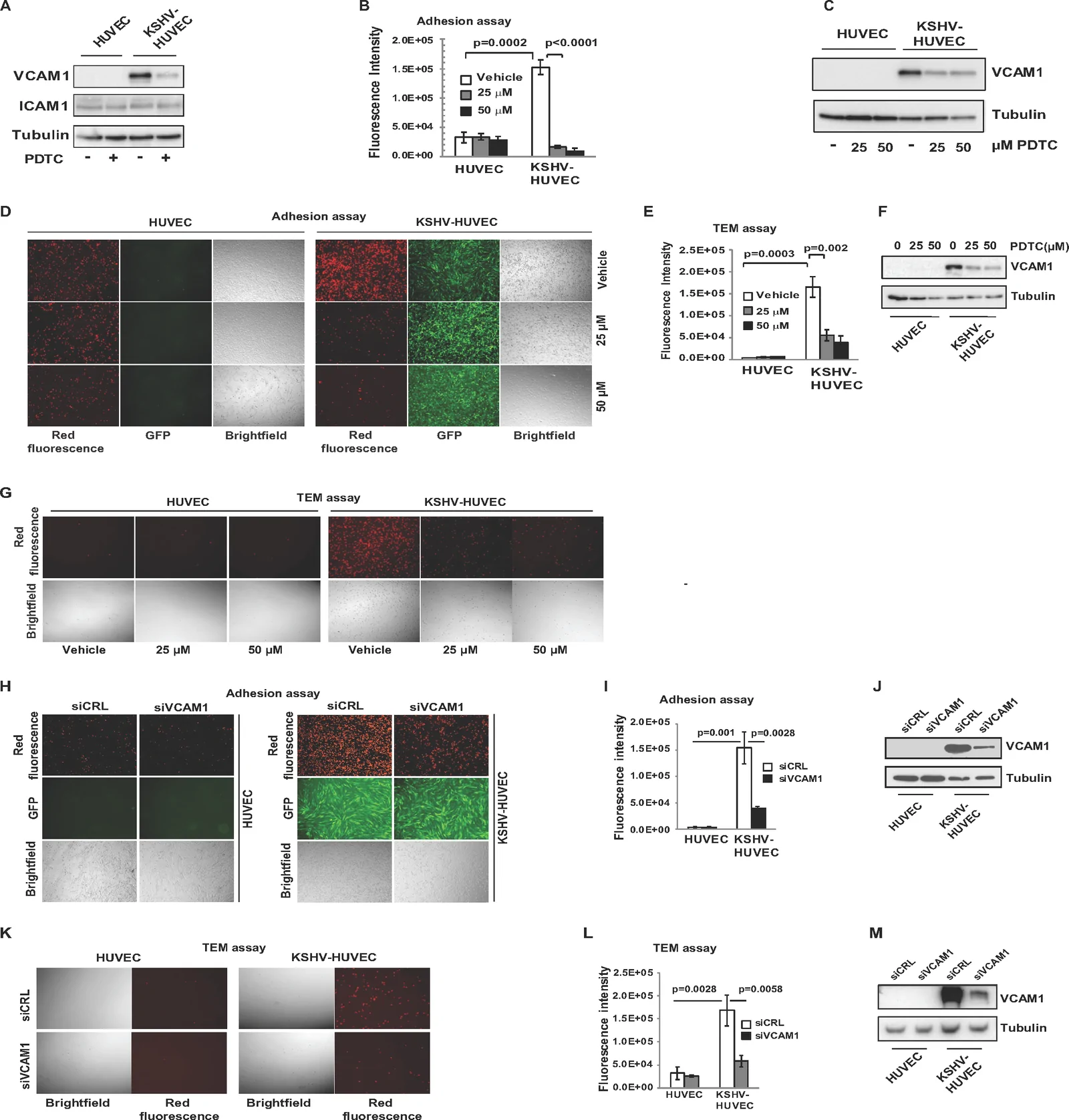
KSHV infection induces VCAM1 expression in endothelial cells and VCAM1-dependent cell adhesion and TEM. (**A**) KSHV-infected and uninfected HUVEC cells treated with 25 μM and 50 µM PDTC for 24 h were subjected to Western blot analysis. The blot was first probed with an anti-VCAM1 antibody, stripped and probed with an anti-ICAM antibody, and subsequently stripped and probed with an anti-tubulin (HRP) antibody. For the adhesion assay, 2 × 10^5^ THP-1 cells stained with CellTrace Far Red dye were added to KSHV-infected and uninfected HUVEC cells which had been treated with PDTC for 24 h and incubated for 6 min for the adhesion assay. Stained THP-1 cells that were adhered to the endothelial cell layer were photographed, and the fluorescence intensity was measured. (**B**) Fluorescence intensity of stained THP-1 cells that were adhered to the endothelial layer was measured on a Clariostar Microplate Reader. (**C**) Western blot analysis was performed with cell lysates from the adhesion assay. After probing with anti-VCAM1 antibody, the blot was stripped and reprobed with anti-tubulin antibody. (**D**) Images at 4× magnification were taken with a fluorescence microscope. (**E**) For the TEM assay, stained THP-1 cells were added to PDTC-treated endothelial cell layer for 4 h; migrating THP-1 cells were collected from the well and washed and photographed; and the fluorescence intensity of the stained THP-1 cells was measured on a Clariostar Microplate Reader. (**F**) Western blot analysis was performed with cell lysates from (**E**). The membrane was first probed with an anti-VCAM1 antibody and then stripped and probed with an anti-tubulin antibody. (**G**) Images of stained THP-1 cells that migrated through the endothelial cell layer were taken at 4× magnification with a fluorescence microscope. (**H**) To deplete VCAM1, KSHV-infected and uninfected HUVEC cells were reverse-transfected with 50 nM of siVCAM1 and control siRNAs for 48 h for the adhesion assay and for 72 h for TEM assay. Images of the stained THP-1 cells that were adhered to the endothelial cell layer were taken at 4× magnification with a fluorescence microscope. (**I**) Fluorescence intensity of stained THP-1 cells adhered to the endothelial cell layer was measured using a Clariostar Microplate Reader. (**J**) Lysates of the endothelial cells collected from the adhesion assay in panel **H** were subjected to Western blot analysis. The membrane was first probed with an anti-VCAM1 antibody and then stripped and probed with an anti-tubulin antibody. (**K**) Images of the stained THP-1 cells that migrated through the endothelial cell layer were taken at 4× magnification with a fluorescence microscope. (**L**) Fluorescent intensity of the stained THP-1 cells that migrated through the endothelial cell layer was measured using the Clariostar Microplate reader. (**M**) Lysates of the endothelial cells from the TEM assay in (**K**) were subjected to Western blot analysis. The membrane was cut into upper and lower sections. The upper section was probed with anti-VCAM1 antibody, and the lower section was probed with anti-tubulin antibody. For panels **B**, **E**, **I**, and **L**, all *P*-values are the result of two-tailed Student’s *t* tests; exact *P*-values are indicated directly within each corresponding figure panel. Error bars indicate the standard error of the means of three independent biological replicates (*n* = 3) for each corresponding panel.

We previously reported that latent KSHV infection increases permeability of human endothelial cells (12). To determine whether KSHV infection affects the expression of adhesion molecules on endothelial cells, we evaluated the expression of VCAM1 and ICAM-1 in lysates of hTert-immortalized HUVEC (referred to as HUVEC) and hTert-immortalized KSHV-infected HUVEC (referred to as KSHV-HUVEC) by SDS-PAGE and Western blot. We found that normal resting KSHV-HUVEC cells express high levels of VCAM1, but we did not detect VCAM1 in uninfected resting HUVEC as previously reported (30) (Fig. 1A). We also evaluated expression levels of the adhesion protein, ICAM-1. We did not detect differences in the levels of ICAM-1 between infected and uninfected endothelial cells (Fig. 1A).

The antioxidant pyrrolidine dithiocarbamate (PDTC) inhibits VCAM1 expression in TNFα-stimulated endothelial cells (31). To determine whether VCAM1 levels are similarly impacted by PDTC in KSHV-infected HUVEC, we treated KSHV-HUVEC with PDTC and evaluated VCAM1 levels by Western blot. We observed that VCAM1 expression is reduced in KSHV-HUVEC following PDTC treatment, but ICAM-1 expression remains unchanged (Fig. 1A). To confirm the upregulation of VCAM1 in different types of endothelial cells, we also assessed VCAM1 levels in *de novo* KSHV-infected primary HUVEC (Fig. S1D and E), stable hTert-immortalized KSHV-infected HMVEC cells (Fig. S1F and G), and primary HMVEC cells (Fig. S1H and I) with Western blot analysis. Infected cells expressed a higher level of VCAM1 compared to uninfected cells.

It has previously been reported that monocytes, eosinophils, basophils, and NK cells use integrin α4β1 to bind VCAM1 on cytokine-activated endothelial cells (32). To determine whether KSHV promotes monocyte adhesion to endothelial cells in a VCAM1-dependent manner, we compared uninfected HUVEC and KSHV-infected HUVEC. The KSHV-infected HUVEC cells express GFP under the control of an EF-1a (human elongation factor-1 alpha) promoter, while uninfected HUVEC do not. We compared the number of THP-1 monocytic cells bound to HUVEC or KSHV-HUVEC in the absence or presence of PDTC using a fluorometric cell adhesion assay (see Materials and Methods). THP-1, a human monocytic cell line, was stained with red CellTrace dye and then incubated with HUVEC or KSHV-HUVEC. After incubating the cells together for 6 min at 37°C, followed by extensive washing, we evaluated the fluorescence intensity of stained THP-1 as a surrogate for THP-1 cell number. We observed a significant increase in red fluorescence intensity when THP-1 cells were incubated with KSHV-HUVEC compared to uninfected HUVEC (Fig. 1B and D). Treatment with 25 µM and 50 μM of PDTC resulted in a reduction of THP-1 cell adhesion to KSHV-HUVEC compared to untreated KSHV-HUVEC by 9- and 16-fold, respectively (Fig. 1B). We found that PDTC treatment also leads to a reduction in VCAM1 levels in the KSHV-HUVEC by Western blot (Fig. 1C). Thus, a reduction in VCAM1 levels on KSHV-HUVEC correlates with reduced THP-1 cell adhesion. We also observed that more THP-1 cells adhered to KSHV-HUVEC vs HUVEC as visualized by fluorescent microscopy of the stained THP-1 cells (Fig. 1D, first row), which was inhibited by PDTC treatment (Fig. 1D). These data suggest that KSHV infection promotes cell adhesion between endothelial and monocytic cells, possibly in a VCAM1-dependent manner.

The interaction of integrin α4β1 on monocytes and VCAM1 on endothelial cells not only facilitates adhesion but also promotes the lateral transendothelial migration of monocytes on endothelial cells and thereby supports TEM (33–35). To evaluate whether KSHV infection affects monocyte transendothelial migration across endothelial cell layers and whether PDTC inhibits TEM, we performed a TEM assay in the presence or absence of PDTC. THP-1 cells stained with red CellTrace stock solution were added to the top of a transwell containing a confluent layer of HUVEC or KSHV-HUVEC cells treated with and without PDTC. Following co-culture, THP-1 cells that had migrated through the endothelial cell layer and were now at the bottom of the transwell were collected and quantified by fluorescence intensity. We observed that monocyte transendothelial migration was significantly higher through the KSHV-HUVEC layer compared to uninfected HUVEC (Fig. 1E). Treatment of KSHV-HUVEC with 25 μM and 50 μM PDTC resulted in a decrease in THP-1 transendothelial migration compared to no PDTC treatment (Fig. 1E). We observed similar trends by fluorescent microscopy. There were more THP-1 cells that underwent transendothelial migration in co-culture with KSHV-HUVEC than with uninfected HUVEC, which was significantly reduced upon PDTC treatment (Fig. 1G). PDTC treatment also led to a reduction in VCAM1 expression, as determined by Western blot (Fig. 1F).

### Expression of VCAM1 is required for enhancement of leukocyte adhesion and transendothelial migration

Our data suggest that KSHV-HUVEC enable THP-1 cell adhesion and TEM, possibly in a VCAM1-dependent manner. Because PDTC is a general antioxidant, we sought to determine specifically whether VCAM1 is required for THP-1 cell adhesion and TEM. We treated KSHV-HUVEC with VCAM1 siRNA to deplete VCAM1 and evaluated THP-1 adhesion. There was a dramatic reduction in THP-1 adherence to KSHV-HUVEC transfected with VCAM1 siRNA compared to cells transfected with a control siRNA by fluorescent microscopy, and this reduction was significant as determined by fluorescent intensity (Fig. 1H and I). As previously observed, there are only low levels of THP-1 cells adhering to uninfected HUVEC, and VCAM1 siRNA treatment did not further impact it (Fig. 1H and I). We confirmed VCAM1 knockdown in the endothelial cells via Western blot (Fig. 1J). To further verify the role of VCAM1 in cell adhesion, we first created a VCAM1 KO HUVEC cell line (VCAM1 KO HUVEC). We next created a FLAG-tagged VCAM1 (rVCAM1_FLAG_-HUVEC) or empty vector (EV-VCAM1 KO HUVEC) cell line. Western blot analysis showed that rVCAM1_FLAG_-HUVEC overexpressed VCAM1 (Fig. S1C). We subsequently performed an adhesion assay which demonstrated that the number of THP-1 cells bound to rVCAM1_FLAG_-HUVEC is significantly higher than that of EV VCAM1 KO HUVEC (Fig. S1A and B).

We next sought to determine whether KSHV-induced VCAM1 expression is important for TEM. To evaluate TEM, we treated KSHV-HUVEC and HUVEC with VCAM1 siRNA for 72 h and conducted the TEM assay. We observed a reduction in THP-1 transendothelial migration with KSHV-HUVEC that had been treated with VCAM1 siRNA compared to KSHV-HUVEC treated with control siRNA by fluorescent microscopy, and this difference was significant following quantification of fluorescence intensity (Fig. 1K and L). We confirmed reduced VCAM1 expression in endothelial cells transfected with VCAM1 siRNA by Western blot (Fig. 1M). These data suggest that THP-1 adhesion and TEM are at least partially mediated by VCAM1 expression on KSHV-HUVEC.

We next used a VCAM1 blocking antibody to determine the importance of surface VCAM1 expression (36–38). We treated KSHV-HUVEC with an anti-VCAM1 antibody or control IgG for 10 min and then added red CellTrace-stained THP-1 cells to the KSHV-HUVEC layer. After several washes, we evaluated the fluorescence intensity of the cells. We noted a significant decrease in THP-1 cell adherence to KSHV-HUVEC following treatment with the anti-VCAM1 antibody compared to control IgG-treated KSHV-HUVEC (Fig. S2A and B). The decrease in THP-1 cell adhesion following anti-VCAM1 antibody treatment suggests that KSHV-induced surface expression of VCAM1 on endothelial cells is important for monocyte adhesion. We also examined TEM by treating KSHV-HUVEC and uninfected HUVEC with the anti-VCAM1 antibody for 15 min and then added stained THP-1 cells. Following co-culture, we evaluated THP-1 cell transendothelial migration using the TEM assay. The addition of the VCAM1 blocking antibody to KSHV-HUVEC significantly reduced THP-1 cell transendothelial migration compared to the control antibody-treated KSHV-HUVEC (Fig. S2C through E). These data indicate that VCAM1 expression is required for both monocytic cell adherence to KSHV-HUVEC and TEM.

### Upregulation of VCAM1 expression in KSHV-HUVEC involves the NF-κB2 pathway members, NIK, IKKα, RelB, and NF-κB p52

**Fig 2:**
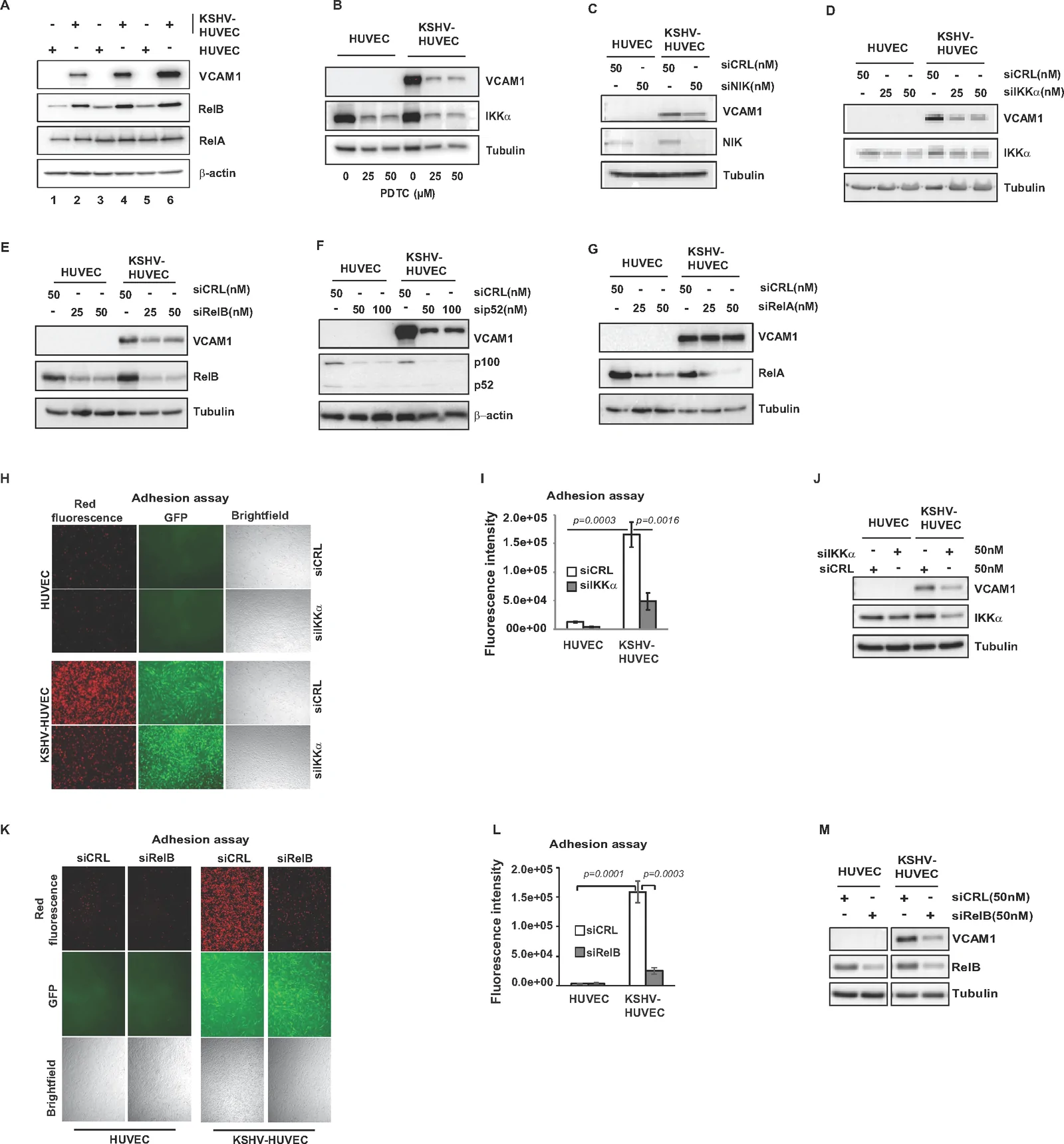
Regulation of VCAM1 involves NFκB2 members, and knockdown of IKKα and RelB inhibits VCAM1-dependent leukocyte adhesion. (**A**) KSHV-infected and uninfected HUVECs were grown for 48 h prior to addition of plain EBM-2 medium (lanes 1 and 2), 10% FBS-EBM-2 (lanes 3 and 4), or 10% FBS-EBM-2 plus supplements (lanes 5 and 6) for 24 h. Cell lysates were harvested for Western blot analysis. The membrane was first probed with an anti-VCAM1 antibody and stripped and probed with anti-RelB, anti-NFκB p65 antibody, and anti-β-actin (HRP) antibodies, respectively. (**B**) KSHV-infected and uninfected HUVECs were grown in 10% FBS-EBM-2 plus supplements and treated with PDTC for 24 h and analyzed by Western blot. The membrane was first probed with an anti-IKKα antibody and then stripped and probed with anti-VCAM1 and anti-tubulin (HRP) antibodies, respectively. KSHV-infected and uninfected HUVECs (2 × 10^6^) were treated with 50 nM siRNAs against NIK (**C**), IKKα (**D**), RelB (**E**), NFκB p52 (**F**), and RelA (**G**) for 48 h, and cell lysates were harvested for Western blot analysis. (**C**) The membrane was first probed with an anti-VCAM1 antibody and then stripped and probed with an anti-tubulin (HRP) antibody and an anti-NIK antibody, respectively. (**D**) The membrane was first probed with an anti-VCAM1 antibody and then stripped and probed with an anti-IKKa antibody and an anti-tubulin (HRP) antibody, respectively. (**E**) The membrane was first probed with an anti-VCAM1 antibody and then stripped and probed with an anti-RelB antibody and an anti-tubulin (HRP) antibody, respectively. (**F**) The membrane was first probed with anti-NF-κB2 p100/p52 antibody and then stripped and probed with anti-VCAM1 antibody and anti-β-actin (HRP) antibody, respectively. (**G**) The transferred membranes were first tested with anti-VCAM1 antibody and then stripped and probed with anti-NF-kB p65 antibody and anti-tubulin (HRP) antibody, respectively. (**H**) KSHV-infected and uninfected HUVEC (2 × 10^5^) per well in a 48-well plate were reverse-transfected with 50 nM of IKKα siRNA for 48 h for the adhesion assay. Stained THP-1 cells were added, and images of the THP-1 cells attached to the endothelial cell layer were taken with a fluorescence microscope. (**I**) Fluorescence intensity of the stained THP-1 cells attached to the siIKKα-treated endothelial cells was measured with a Clariostar Microplate Reader. (**J**) Western blot analysis of cell lysates of the siIKKα-treated endothelial cells. The membrane was first probed with anti-VCAM1 antibody and then stripped and probed with anti-IKKα antibody and anti-tubulin (HRP) antibody, respectively. (**K**) Images of the stained THP-1 cells attached to the siRelB-treated endothelial cell layer were taken with a fluorescence microscope. (**L**) Fluorescence intensity of the stained THP-1 cells attached to the siRelB-treated endothelial cell layer was measured with a Clariostar Microplate Reader. (**M**) Western blot analysis of siRelB-treated endothelial cells. The membrane was first probed with an anti-VCAM1 antibody and then stripped and probed with anti-RelB antibody and anti-tubulin (HRP) antibody, respectively. All *P*-values of panel **I** and **L** are the result of two-tailed Student’s *t* tests; exact *P*-values are indicated directly within each corresponding figure panel. Error bars represent the standard error of the means of three independent biological replicates (*n* = 3) for each corresponding panel.

KSHV infection is known to induce NF-κB (39). Because we observed an increase in VCAM1 expression in KSHV-HUVEC and its expression is important for THP-1 cell adhesion and TEM, we next determined the mechanism by which KSHV-HUVEC upregulates VCAM1. The NF-ĸB pathway includes canonical (NF-κB1) and non-canonical (NF-κB2) pathways, which commonly involve NF-κB RelA (p65)/p50 and RelB/p52, respectively. To determine NF-кB pathway members that were involved in upregulating VCAM1 in KSHV-HUVEC because of KSHV infection rather than serum, KSHV-HUVEC and HUVEC were grown in different serum conditions, and the expression levels of RelB and RelA were evaluated by Western blot. We found that KSHV infection increased the level of RelB and VCAM1 (Fig. 2A, lanes 2, 4, and 6), but not RelA, compared to uninfected HUVEC (Fig. 2A, lanes 1, 3, and 5) even in the absence of serum and supplements. These data suggest that NF-ĸB2 members may be involved in regulating VCAM1 expression in KSHV-HUVEC. PDTC is also an inhibitor of the NF-ĸB pathway (31, 40, 41). We treated KSHV-HUVEC and HUVEC with PDTC for 24 h and observed reduced expression of IKKα in KSHV-HUVEC and HUVEC (Fig. 2B). As previously observed, VCAM1 expression was also reduced in PDTC-treated KSHV-HUVEC (Fig. 2B). These data suggest that increased VCAM1 expression in KSHV-HUVEC may involve the NF-кB2 pathway. To confirm that the upregulation of VCAM1 expression in KSHV-HUVEC involves the NF-ĸB2 pathway, we evaluated expression levels of VCAM1 and NF-кB2 members following knockdown of NIK, IKKα, RelB, and NF-ĸB p52 in KSHV-HUVEC. We found that VCAM1 expression in KSHV-HUVEC was inhibited by the depletion of NIK, IKKα, RelB, and NF-ĸB p52 (Fig. 2C through F). These data suggest that the non-canonical NF-ĸB pathway is involved in KSHV-induced upregulation of VCAM1. In contrast, the induction of VCAM1 is independent of RelA (Fig. 2G).

### Leukocyte cell adhesion to KSHV-infected endothelial cells is dependent on the NF-кB2 pathway

To determine whether leukocyte adhesion to KSHV-HUVEC is dependent on the NF-кB2 pathway, we evaluated THP-1 cell adhesion following knockdown of non-canonical NF-кB family members including IKKα, RelB, NF-ĸB p52, and NIK in KSHV-HUVEC. We observed that depletion of IKKα significantly abrogates THP-1 cell adherence to IKKα-siRNA-treated KSHV-HUVEC (Fig. 2H and I) and that VCAM1 expression in KSHV-HUVEC was reduced upon IKKα knockdown (Fig. 2J).

**Fig 3:**
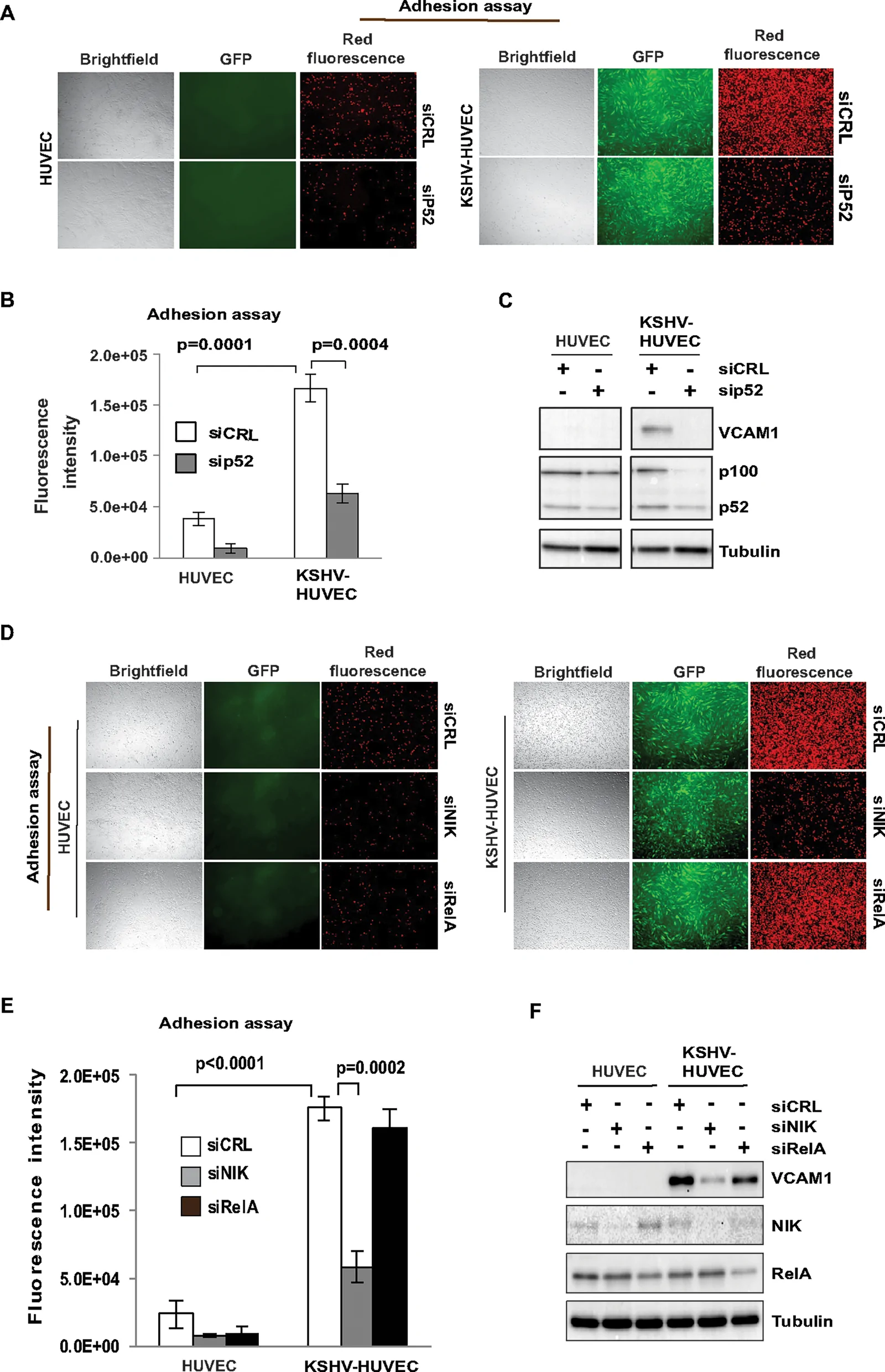
Knockdown of NFκB p52 and NIK impairs VCAM1-dependent cell adhesion. Cell adhesion assay of KSHV-infected and uninfected HUVECs was conducted following treatment with 50 nM of siRNAs of NFκB p52 (**A–C**), NIK (**D–F**), or RelA (**D–F**). (**A**) KSHV-infected and uninfected HUVEC (2 × 10^5^) were reverse-transfected with 50 nM of p52 siRNA for 48 h. Stained THP-1 cells were added, and images of the THP-1 cells attached to the sip52-treated endothelial cells were taken with a fluorescence microscope. (**B**) Fluorescent intensity of the stained THP-1 cells attached to the sip52-treated endothelial cell layer was taken with a Clariostar Microplate Reader. (**C**) Lysates of the sip52-treated endothelial cells were subjected to Western blot analysis. The membrane was first probed with an anti-NF-κB2 p100/p52 antibody, and then the blot was stripped and probed with anti-VCAM1 antibody and anti-tubulin (HRP) antibody, respectively. (**D**) KSHV-infected and uninfected HUVEC (2 × 10^5^) were reverse-transfected with 50 nM NIK or RelA siRNA for 48 h for the adhesion assay. Stained THP-1 cells were added, and images of the THP-1 cells attached to the siNIK- or siRelA-treated endothelial cells were taken with a fluorescence microscope. (**E**) Fluorescent intensity of the stained THP-1 cells adhered to the siNIK- or siRelA- treated endothelial cells was measured with a Clariostar Microplate Reader. (**F**) Lysates of the siNIK- and siRelA-treated endothelial cells were subjected to Western blot analysis. The membrane was first probed with an anti-NIK antibody. The blot was then stripped and probed with an anti-VCAM1 antibody, anti-NF-κB p65 antibody, and anti-tubulin (HRP) antibody, respectively. All *P*-values of panel **B** and **E** are results of two-tailed Student’s *t*-tests; exact *P*-values are indicated directly within each corresponding figure panel. Error bars represent the standard error of the means of three independent biological replicates (*n* = 3) for each corresponding panel.

We also determined whether VCAM1-dependent THP-1 adhesion is impacted by RelB. We treated KSHV-HUVEC and HUVEC with RelB siRNA and evaluated THP-1 cell adhesion. We noted a strong suppression of cell adhesion following depletion of RelB in KSHV-HUVEC (Fig. 2K and L). Upon RelB knockdown, VCAM1 levels were also reduced (Fig. 2M). Similarly, knockdown of NF-ĸB p52 and NIK resulted in reduced THP-1 cell adherence to KSHV-HUVEC (Fig. 3A, B, D, and E). VCAM1 protein levels were also reduced in NF-ĸB p52 (Fig. 3C) and NIK (Fig. 3F) siRNA-treated KSHV-HUVEC compared to control siRNA-treated KSHV-HUVEC. In contrast, THP-1 cell adhesion to KSHV-HUVEC treated with RelA siRNA compared to the control siRNA remained unchanged (Fig. 3D and E); correspondingly, RelA knockdown did not alter VCAM1 expression (Fig. 3F). These data strongly suggest that KSHV infection of endothelial cells increases VCAM1 via the NF-кB non-canonical pathway, thereby enhancing leukocyte adhesion.

### Leukocyte transendothelial migration is dependent on NF-кB2 family members in KSHV-HUVEC

**Fig 4:**
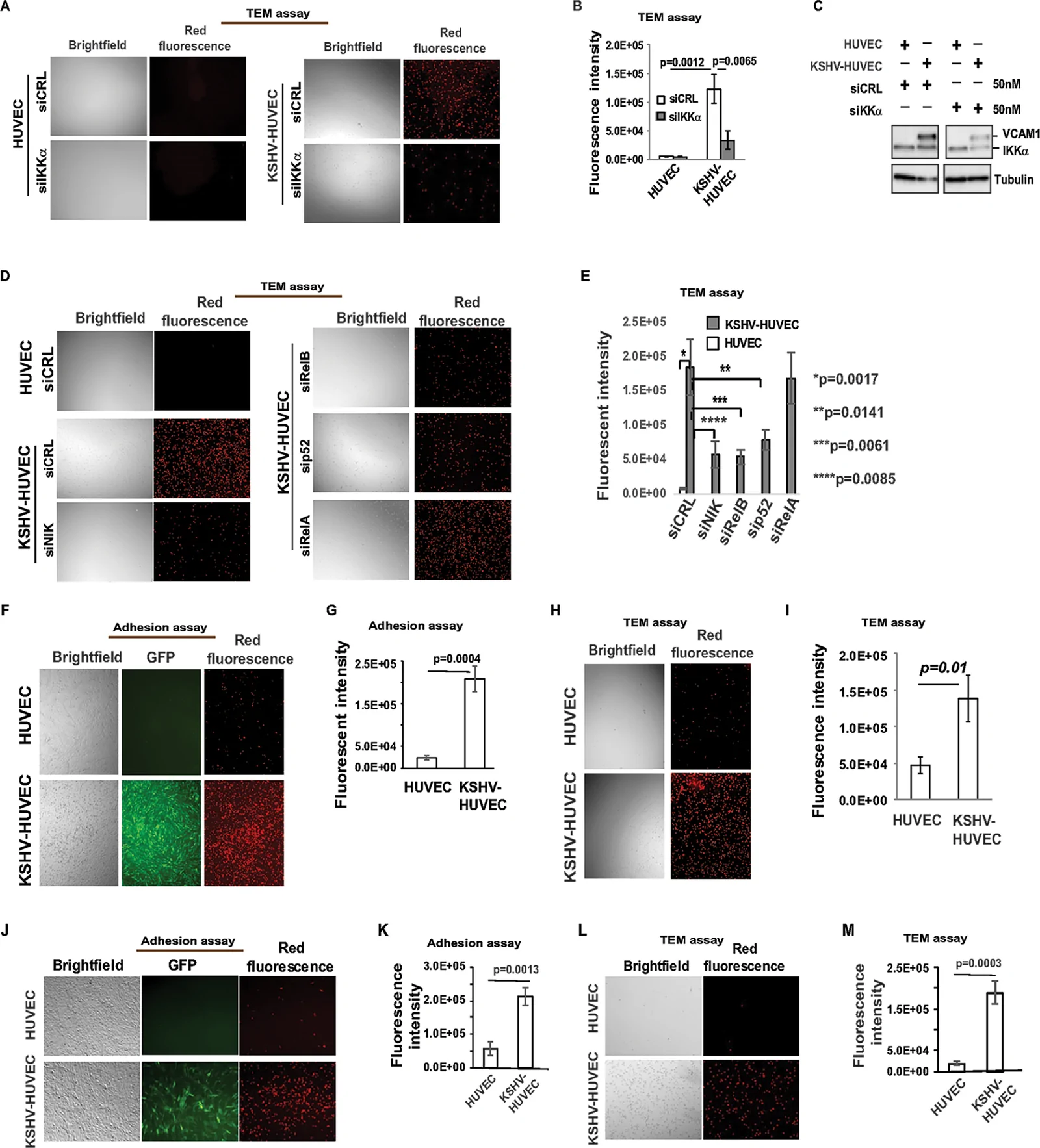
Knockdown of IKKα, NIK, RelB, and NFĸB p52 results in decreased transendothelial migration, and KSHV infection enhances primary leukocyte adhesion and transendothelial migration. KSHV-infected and uninfected HUVEC (3 × 10^5^) were reverse-transfected with 50 nM of IKKα siRNA (**A–C**), or NIK, RelB, NFkB2 p52, and RelA siRNA (**D and E**) for 72 h. Next, 2 × 10^5^ THP-1 cells stained with CellTrace Far Red dye were added and incubated at 37°C, 5% CO_2_ for 4 h for the TEM assays. Migrating THP-1 cells were collected, washed, and photographed, and fluorescence intensity was measured using a fluorescence microscope and a Clariostar Microplate Reader. (**A**) Images of stained THP-1 cells that migrated through the siIKKα-treated endothelial cell layer are depicted. (**B**) Fluorescent intensity of the stained THP-1 cells that migrated through the siIKKα-treated endothelial cell layer was measured with a Clariostar Microplate reader. (**C**) The siIKKα-treated endothelial cells were harvested and subjected to Western blot analysis. The membrane was first probed with anti-VCAM1 and anti-IKKα antibodies and then stripped and probed with anti-tubulin (HRP) antibody. (**D**) Images of the stained THP-1 cells that migrated through the siNIK-, siRelB-, sip52-, or siRelA-treated endothelial cell layer were taken with a fluorescence microscope as shown. (**E**) Fluorescent intensity of the stained THP-1 cells that migrated through the siNIK-, siRelB-, sip52-, or siRelA-treated endothelial cell layer was measured with a Clariostar Microplate reader. (**F**) KSHV-infected and uninfected HUVEC (1 × 10^5^) were plated per well of a 48 well plate and allowed to form a monolayer. Human primary monocytes (3 × 10^5^) stained with CellTrace Far Red dye were added to the endothelial cell layer and incubated for 35 min for the adhesion assay, and images of the stained primary monocytes attached to the endothelial cell layer were taken with a fluorescence microscope. (**G**) Fluorescent intensity of the stained primary monocytes that were adhered to the endothelial cell layer was measured with a Clariostar Microplate reader. (**H**) KSHV-infected and uninfected HUVEC (2 × 10^5^) were plated per insert allowed to form a layer; 3 × 10^5^ human primary monocytes stained with CellTrace Far Red dye were added to the endothelial cell layer and incubated for 7 h for the TEM assay; images of the stained primary monocytes that migrated through the endothelial cell layer were taken with a fluorescence microscope. (**I**) Fluorescent intensity of the stained primary monocytes that migrated through the endothelial cell layer was measured with a Clariostar Microplate reader. (**J**) Human primary B cells (1.3 × 10^6^) stained with CellTrace Far Red dye were added onto the KSHV-infected and uninfected HUVEC cell layer for 3 h in the cell adhesion assay. Images of stained primary B cells (10× magnification) adhered to the endothelial cell layer were taken with a fluorescence microscope. (**K**) Fluorescent intensity of the stained primary B cells adhered to the endothelial cell layer was measured with a Clariostar Microplate reader. (**L**) Human primary B cells (1.3 × 10^6^) stained with CellTrace Far Red dye were added onto the KSHV-infected and uninfected HUVEC cell layer for 8 h in the TEM assay. Images of stained primary B cells (10× magnification) that migrated through the endothelial cell layer were taken with a fluorescence microscope. (**M**) Fluorescent intensity of the stained primary B cells that migrated through the endothelial cell layer was measured with a Clariostar Microplate reader. For panels **B**, **E**, **G, I**, **K**, and **M**, all *P*-values are the result of two-tailed Student’s *t*-tests; exact *P*-values are indicated directly within each corresponding figure panel. The error bars represent the standard error of the means of three independent biological replicates (*n* = 3) for each panel.

We next evaluated whether THP-1 TEM is dependent on the NF-кB2 pathway. We treated KSHV-HUVEC and HUVEC with siRNA directed against IKKα, NIK, RelB, or NFĸB p52 and then assessed leukocyte TEM. We observed reduced THP-1 cell TEM in KSHV-HUVEC that had been treated with IKKα siRNA (Fig. 4A and B; Fig. S3A). We confirmed that IKKα and VCAM1 levels are reduced in KSHV-HUVEC treated with IKKα siRNA compared to control siRNA (Fig. 4C). We then systematically evaluated THP-1 cell TEM in KSHV-HUVEC that had been treated with siRNA against NIK, RelB, or NF-ĸB p52. As with IKKα depletion, knockdown of NIK, RelB, or NF-кB p52 resulted in a reduction of THP-1 TEM (Fig. 4D and E; Fig. S3B).

We also evaluated THP-1 cell TEM in KSHV-HUVEC that had been treated with siRNA against the NF-кB canonical pathway member, RelA. We found that THP-1 cell TEM was not modulated by RelA knockdown and the canonical NF-кB pathway (Fig. 4D and E; Fig. S3B).

### Increased adhesion and TEM of primary leukocytes co-cultured with KSHV-HUVEC

To further assess cell adhesion and transendothelial migration of primary leukocytes across KSHV-infected endothelial cell layers, we isolated primary monocytes and primary B cells from three healthy human donors for each cell type. We incubated these red Celltrace dye-stained primary cells with KSHV-HUVEC or HUVEC and evaluated adhesion by measuring fluorescence intensity. As in the case with the THP-1 cells, we noted a significant increase in primary monocyte (Fig. 4F and G) and primary B cell (Fig. 4J and K; Fig. S4A) adherence to KSHV-HUVEC compared to uninfected HUVEC as indicated by an increase in fluorescence intensity and cell numbers. We then evaluated transendothelial migration of primary monocytes and B cells in the presence of KSHV-HUVEC or HUVEC using the TEM assay. We observed an increase in TEM of primary monocytes (Fig. 4H and I; Fig. S3C) and primary B cells (Fig. 4L and M; Fig. S4B) when co-cultured with KSHV-HUVEC compared to HUVEC. These data support the overall notion that KSHV infection enhances adhesion and TEM of leukocytes, which may explain the fact that KS lesions contain infiltrating immune cells.

### VCAM1 is expressed in KSHV-infected tumors

**Fig 5:**
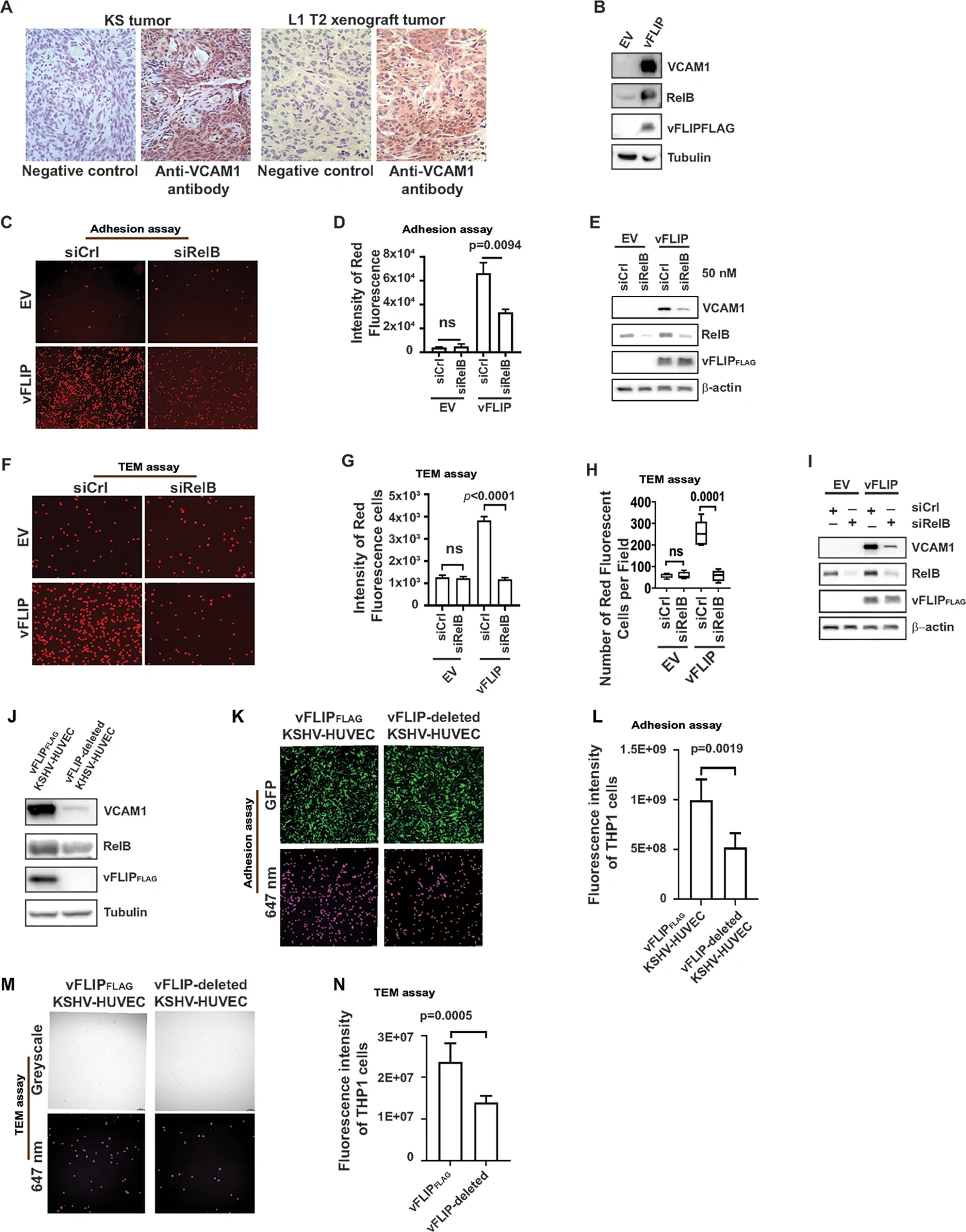
KS tumors express VCAM1, and KSHV vFLIP enhances THP-1 adhesion and TEM via induction of VCAM1. (**A**) For immunohistochemistry, slides of L1T2 xenograft tumor and slides of human KS tumor were deparaffinized using Histochoice and quenched with 3% H_2_O_2_ prior to the addition of anti-VCAM1 antibody and blocking buffer as negative control. Images were taken at 400× magnification with a Leica DM LA histology microscope after incubation with anti-rabbit secondary antibody and staining with vector peroxidase substrate. (**B**) FLAG-tagged vFLIP-expressing lentivirus and empty vector lentivirus were transduced into uninfected HUVEC to establish a stable vFLIP-expressing HUVEC (vFLIP-HUVEC) cell line and an empty vector HUVEC (EV-HUVEC) stable cell line. Lysates of vFLIP-HUVEC and EV-HUVEC were subject to Western blot analysis. The membrane was first probed with anti-VCAM1 and anti-RelB antibodies, and then the blot was stripped and probed with anti-FLAG (HRP) and anti-tubulin (HRP) antibodies, respectively. (**C**) For the adhesion assay, vFLIP-HUVEC and EV-HUVEC were reverse-transfected with 50 nM of control siRNA (siCrl) and RelB siRNA for 72 h. THP-1 cells stained with CellTrace Far Red dye were added to the layer of vFLIP-HUVEC and EV-HUVEC for 6 min at 37°C and 5% CO_2_. The cells were washed several times, and images were taken at 10× magnification with a fluorescent microscope. (**D**) Fluorescent intensity of the stained THP-1 cells adhered to the endothelial cell layer was measured with a Clariostar Microplate reader. (**E**) Lysates of the endothelial cells were subjected to Western blot analysis. The membrane was first probed with an anti-VCAM1 antibody, and then the blot was stripped and probed with anti-RelB, anti-FLAG (HRP), and anti-β-actin (HRP) antibodies, respectively. (**F**) For the TEM assay, 3 × 10^5^ vFLIP-HUVEC and EV-HUVEC were subjected to reverse transfection with siCrl and siRelB. Stained THP-1 cells were added to the layer of vFLIP-HUVEC or EV-HUVEC and incubated for 5 h and 30 min at 37°C and 5% CO_2_. The stained cells were collected and washed multiple times. Images of the stained THP-1 cells were taken at 10× magnification with a fluorescence microscope. (**G**) The intensity of the stained THP-1 cells that had migrated through the endothelial cell layer was measured with a Clariostar Microplate reader. (**H**) The number of stained THP-1 cells that migrated through the vFLIP-HUVEC or EV-HUVEC layer was counted under a fluorescent microscope. (**I**) Lysates of the endothelial cells from (**G and H**) were subjected to Western blot analysis. The membrane was first probed with an anti-VCAM1 antibody, and then the blot was stripped and probed with anti-RelB, anti-FLAG (HRP), and anti-β-actin (HRP) antibodies, respectively. (**J**) WT vFLIP_FLAG_ KSHV- HUVEC and vFLIP-deleted KSHV-HUVEC lines were established from iSLK.BAC16-vFLIP_FLAG_ and iSLK.BAC16-vFLIP KO as described in the Materials and Methods. Lysates of WT vFLIP_FLAG_ KSHV-HUVEC and vFLIP-deleted KSHV-HUVEC were subject to Western blot analysis. The membrane was first probed with an anti-VCAM1 antibody, and then the blot was stripped and probed with anti-RelB, anti-FLAG (HRP), and anti-tubulin (HRP) antibodies, respectively. (**K**) Stained THP-1 cells were added to the layer of WT vFLIP_FLAG_ KSHV-HUVEC and vFLIP-deleted KSHV- HUVEC cells for the adhesion assay, and images were taken with a fluorescence microscope. (**L**) Fluorescence intensity of stained THP-1 cells adhered to the endothelial cells was measured. (**M**) Stained THP-1 cells were added to the layer of WT vFLIP_FLAG_ KSHV-HUVEC and vFLIP-deleted KSHV-HUVEC cells for the TEM assay, and images of the stained THP-1 cells that migrated through the endothelial cell layer were taken with a fluorescence microscope. (**N**) Fluorescence intensity of stained THP-1 cells that had migrated through the endothelial cell layer was measured. All *P*-values of panels **D**, **G**, **H**, **L**, and **N** are the result of two-tailed Student’s *t*-tests; exact *P*-values are indicated directly within each corresponding figure panel. The error bars represent the standard error of the means of three independent biological replicates (*n* = 3) for each panel.

Although we found that KSHV-infected endothelial cells express high levels of VCAM1 compared to uninfected cells, we wanted to determine whether VCAM1 is expressed in KSHV-infected tumors. Thus, we evaluated VCAM1 expression in L1T2 xenografts and human KS tumors via immunohistochemistry. L1T2 xenografts are derived by injecting KSHV-infected TIVE cells (L1T2) into nude mice (42). Compared to the negative controls, we found that VCAM1 is, indeed, expressed in both L1T2 xenografts and KS tumors (Fig. 5A). This is consistent with prior reports (8, 43).

### KSHV vFLIP enhances adhesion and transendothelial migration via induction of VCAM1

The KSHV vFLIP protein is expressed in KSHV latently infected cells (44), and vFLIP is known to induce the expression of VCAM1 and COX-2 via NFκB signaling (45–47). To test whether vFLIP enhances cell adhesion and transendothelial migration via induction of RelB and via induction of VCAM1, a FLAG-tagged vFLIP-expressing lentivirus was transduced into uninfected HUVEC to establish a stable vFLIP-expressing HUVEC cell line. Additionally, a HUVEC stable line with just empty vector (EV-HUVEC) was also established. Western blot analysis showed that vFLIP expression induced VCAM1 and RelB in HUVEC (Fig. 5B). In addition, the expression of vFLIP increased THP-1 cell adhesion to the vFLIP-expressing HUVEC, which was inhibited by knockdown of RelB (Fig. 5C through E). Figure 5C shows that more stained THP-1 cells adhered to vFLIP-expressing cells than to EV-HUVEC. The intensity of red fluorescent THP-1 cells binding to vFLIP-expressing cells transfected with the control siRNA (siCrl) is 16-fold higher than those bound to EV-HUVEC transfected with the control siRNA (Fig. 5D). Knockdown of RelB caused a reduction of VCAM1 expression (Fig. 5E) and a subsequent decrease of THP-1 adhesion to vFLIP-HUVEC cells (Fig. 5D) compared to control siRNA-treated vFLIP-HUVEC. Next, a transendothelial migration assay was performed. We found that there was an increase of THP-1 cells that transmigrated through the vFLIP-expressing cells (vFLIP-HUVEC) compared to EV-HUVEC (Fig. 5F through H). The number of THP-1 cells transmigrating through the vFLIP-HUVEC was 4.3-fold higher than that of EV-HUVEC (Fig. 5H). When RelB was depleted in the vFLIP-HUVEC using RelB siRNA, it reduced VCAM1 expression (Fig. 5I) and resulted in a lower number of THP-1 cells that could transmigrate through the vFLIP-HUVEC layer compared to cells treated with the control siRNA (Fig. 5G and H).

### A KSHV vFLIP deletion virus reduces VCAM1 expression, leukocyte adhesion, and transendothelial migration

To further confirm whether vFLIP in KSHV-infected cells induces VCAM1 expression and promotes THP-1 adhesion and transendothelial migration, we used a KSHV recombinant virus expressing a FLAG-tagged version of vFLIP or a recombinant virus deleted for vFLIP. We established WT vFLIP_FLAG_ KSHV-HUVEC and vFLIP-deleted KSHV-HUVEC that were derived from iSLK.BAC16-vFLIP_FLAG_ and iSLK.BAC16-vFLIP KO (48) (kind gifts from Dr. Jae Jung). Western blot analysis demonstrated that VCAM1 and RelB are induced in WT vFLIP_FLAG_ KSHV-HUVEC compared to vFLIP-deleted KSHV-HUVEC (Fig. 5J; Fig. S5A and B). Additionally, we compared THP-1 cell adhesion and TEM for WT vFLIP_FLAG_ KSHV-HUVEC to vFLIP-deleted KSHV-HUVEC cells. We observed significantly more THP-1 cells bound to WT vFLIP_FLAG_ KSHV-HUVEC compared to the vFLIP-deleted KSHV-HUVEC (Fig. 5K and L). We next performed a TEM assay. The number of THP-1 cells that transmigrated across the vFLIP-deleted KSHV-HUVEC endothelial cell layer was lower than for the WT vFLIP_FLAG_ KSHV-HUVEC endothelial layer suggesting that the presence of vFLIP in KSHV-infected endothelial cells is important for TEM (Fig. 5M and N). To further confirm whether the regulation of VCAM1 expression involves the NFκB2 pathway, WT vFLIP_FLAG_ KSHV-HUVEC cells were treated with siRelB. We observed that knockdown of RelB decreases VCAM1 expression (Fig. S6D). We further confirmed that THP-1 cell adhesion (Fig. S6A through D) and TEM (Fig. S6E through H) were impacted by knockdown of RelB and VCAM1 in WT vFLIP_FLAG_ KSHV-HUVEC.

## DISCUSSION

In this study, we demonstrate that latent KSHV infection promotes leukocyte adhesion and TEM through vFLIP-dependent induction of VCAM1. This mechanism is particularly relevant because KS lesions typically consist of endothelial-lineage spindle cells and are marked by heavy leukocyte infiltration, including monocytes and B cells (7–11, 49–51). This inflammation and leukocyte infiltration are thought to actively promote KS pathogenesis (8, 51). For instance, KSHV infection has been shown to induce 5-lipoxygenase and its chemotactic metabolite leukotriene B4 to attract monocytes to the site of infection (49). Moreover, leukocytes in the KS lesion may become infected and expand the viral reservoir as KSHV-infected monocytes are found in KS lesions (9, 50, 51). In this paper, we explored the mechanisms by which KSHV-infected endothelial cells promote monocyte adhesion and transendothelial migration. We found that KSHV promotes VCAM1-dependent leukocyte transendothelial migration.

Previous studies showed that the two NF-κB sites in the VCAM1 promoter are crucial for the stimulation of VCAM1 gene transcription by the NF-κB family member p65/p50 complex responding to cytokine stimulation, such as lipopolysaccharide, IL-1β, and TNF-α. However, even with mutations at both NF-κB sites, cytokines can still activate VCAM1 transcription. This suggests the existence of other regulatory mechanisms. In addition, IkB/MAD-3 can inhibit transcription activation of VCAM1 by p65 (22, 52, 53). We found that latent KSHV infection of endothelial cells promotes leukocyte adhesion and transendothelial migration of these monocytes by inducing VCAM1 expression via the NF-кB2 pathway. We also show that VCAM1 is expressed in KS lesions. By expressing VCAM1, KSHV-infected spindle endothelial cells promote the adhesion and transendothelial migration of leukocytes like monocytes through the endothelial cell layer. Prior reports found that KSHV infection of cytokine-activated endothelial cells specifically inhibited neutrophil but not monocyte transendothelial migration (54).

Expression of the latent protein vFLIP has been shown to activate the NF-κB2 pathway (55). Moreover, expression of KSHV vFLIP also results in the spindle shape (47). We further found that expression of VCAM1, induced by vFLIP, increased THP-1 cell adhesion and transendothelial migration. Moreover, VCAM1 expression induced by overexpression of vFLIP involves the NF-кB2 family member, RelB. In addition, we further confirmed that latent KSHV-vFLIP_FLAG_-infected HUVEC expresses vFLIP and increases THP-1 adhesion and transendothelial migration. The KS lesion and the blood of KS patients contain a variety of cytokines (IL6, TNFα, and IL10 [56, 57]) that are known to activate endothelial cells and to stimulate VCAM1 expression (58–62). Most likely, there are multiple mechanisms that lead to VCAM1 expression in KSHV-infected HUVEC but vFLIP is certainly involved in this process. In addition, we found that latent KSHV infection of HMVEC and *de novo* infection of primary HUVEC and primary HMVEC also induce VCAM1 expression.

KSHV infection constitutively activates the transcription factor nuclear factor-kappa B (NF-κB) in endothelial cells and lymphocytes, and activation of NF-κB is crucial for maintenance of the latent viral life cycle (39, 63–65). We observed that induction of VCAM1 by latent KSHV infection involves regulation of NF-κB2 members NIK, IKKα, RelB, and NF-κB p52 but did not involve the NF-кB1 member RelA. In summary, KSHV infection of endothelial cells contributes to upregulation of VCAM1 through vFLIP, thereby allowing for leukocyte adhesion and transendothelial migration. It has previously been reported that the NF-kB pathway is important for upregulating VCAM1 gene expression at the transcriptional level (66).

In summary, our study establishes that vFLIP and NF-κB2 are involved in upregulating VCAM1 expression in endothelial cells and that VCAM1 drives leukocyte adhesion and TEM. It is possible that VCAM1 may also serve as a therapeutic target to impair the leukocyte-driven tumor microenvironment necessary for KS pathogenesis.

## MATERIALS AND METHODS

### Cell culture

The human monocytic cell line (THP-1) (67) was maintained in RPMI 1640 medium (Corning) containing 0.05 mM β-mercaptoethanol. Primary human umbilical vein endothelial cells and immortalized human umbilical vein endothelial cells (hTERT-HUVEC) and KSHV latently infected hTERT-HUVEC (KSHV-HUVEC) (68) were cultured in endothelial growth medium EBM-2 (Lonza) with the EBM-2 bullet supplement (Lonza) at 37°C in a 5% CO_2_ environment. KSHV-HUVEC were maintained in the presence of 0.5 µg/mL of puromycin. Primary human microvascular endothelial cells (ATCC) (pHMVEC), immortalized human microvascular endothelial cells (hTERT-HMVEC) and KSHV latently infected hTERT-HMVEC (KSHV-HMVEC) were cultured in endothelial growth medium EBM-2 (Lonza) and EGM-2 MV supplements (Lonza) at 37°C, 5% CO_2_. Stable KSHV-HMVEC were maintained in the presence of 2 µg/mL of puromycin. THP1 medium contained 10%–15% of FBS. All other media were supplemented with 10% FBS, 1% penicillin-streptomycin, and 1% L-glutamine.

### Virus preparation

A total of 1.2 × 10^7^ iSLK.219 cells were plated in a T175 flask for 24 h and then were reactivated with 1 mM of sodium butyrate and 2 μg/mL doxycycline in DMEM with 2% FBS for 64 h. The supernatant was centrifuged at 950 × *g* for 15 min at 4°C and then filtered through a 0.45-μm filter on ice. The filtered supernatant was poured into Beckman Ultra-Clear centrifuge tubes and 5 mL of 20% sucrose was slowly underlayed in the bottom of the tube. The tube was centrifuged at 24,000 rpm (98,400 × *g*) at 4°C for 2 h and 30 min in Beckman Optima XPN-100. The supernatant and sucrose were removed, and the pellet was suspended in 1× PBS in concentration ratio at 1:200 and stored at −80°C freezer.

### Virus infection

The concentrated KSHV virus stock solution was diluted with EBM-2 basal medium at a ratio of 1:2 (0.2 mL:0.4 mL), and polybrene was added to a final concentration of 8 μg/mL. This virus solution was added to one well of a 24-well plate containing primary HUVECs and primary HMVECs at 90% confluency. The plate was centrifuged at 2,500 rpm for 90 min at 30°C. The supernatant was removed daily, and fresh medium was added to the cells for 5 days. Cell lysates were collected for Western blot analysis.

### Reagents and antibodies

1-Pyrrolidinecarbodithioic acid ammonium salt (PDTC) was purchased from Sigma-Aldrich. Monocyte chemoattractant protein-1 (MCP-1) was obtained from R&D Systems. Antibodies against ICAM1 were obtained from Santa Cruz Biotechnology, and VCAM1, IKKα, RelB, NF-κB1 p65 (RelA), NIK, and NF-κB2 p100/p52 antibodies were from Cell Signaling Technology. Mouse anti-human VCAM1/CD106 blocking monoclonal (1.G11B1) antibody was purchased from LifeSpan BioSciences, and normal mouse IgG (200 µg/500 µL) was from Santa Cruz Biotechnology.

### Plasmid construction

Plenti-CMV-MCS-RFP-SV-puro, a gift from Jonathan Garlick & Behzad Gerami-Naini (Addgene plasmid no. 109377; https://www.addgene.org/109377/; RRID: Addgene 109377), was digested with XbaI and BamHI to remove the DsRed2 gene. The vFLIP gene was amplified from BAC16 by PCR with primers: forward primer, CTCATCTAGAATGGCCACTTACGAGGTTCTC; reverse primer, CGATGGATCCCTACTTGTCGTCGTCATCCTTGTAGTCCTTGTCGTCGTCATCCTTGTAGTCTGGTGTATGGCGATAGTGTTG. The PCR product of Xbal-vFLIP-2XFLAG-BamHI, which was digested with Xbal and BamHI, was inserted into the digested plasmid to produce plenti-CMV-MCS-vFLIP_2XFLAG_, which has a 2×FLAG epitope tag at the C-terminal of vFLIP. plenti-CMV-MCS-vFLIP_2XFLAG_ was used to make lentivirus to infect hTERT-hygromycin HUVEC to build a vFLIP-expressing cell line, and plenti-CMV-MES-2XFLAG was used as an empty vector control.

To construct lentiCRISPR-sgVCAM1, the following protocol was used. gRNA primers oligo-F, 5′ CACCG*CGGTCAAGGGGGTACACGCT*, and oligo-R, 5′ AAAC*AGCGTGTACCCCCTTGACCG**,* were annealed on a heat block at 94°C for 4 min and then incubated at 70°C for 10 min and slowly cooled to room temperature. Plasmid lentiCRISPR-V2 Blast, a gift from Brett Stringer’s lab (Addgene plasmid no. 98293; https://www.addgene.org/98293), was digested with smBI-v2 (NEB, R0739A), and then double-stranded oligo inserts were annealed into the BsmBI-v2 site of lentiCRISPR-V2 to construct lentiCRISPR-sgVCAM1 plasmid. lentiCRISPR-sgVCAM1 was used to make lentivirus with the Viral Power kit (Invitrogen, cat. no. K497500) in 293 FT cells. Lentivirus-sgVCAM1 was transduced into hTERT-hygromycin HUVEC and selected with 10 µg/mL of blasticidin to get VCAM1 KO HUVEC. Lentivirus made with LentiCRISPR-V2 was transduced to HUVEC to build the sgCrl HUVEC cell line.

To construct PLENTI-CMV-MES-2XFLAG and PLENTI-CMV-VCAM1-2XFLAG plasmids, the following protocol was used. To insert a fragment of multiple enzyme sites following 2× FLAG tag into XbaI and BamHI sites of plenti-CMV-MCS-RFP-SV-PURO (Addgene plasmid no. 109377), oligo1 (CATGTCTAGAACCGGTGATATCCTCGAGGGGCCCGACTACAAGGATGACGACGACAAGGACTACAAGGATGACGACGACAAGTGAGGATCCATCG) and its complement oligo 2 (CGATGGATCCTCACTTGTCGTCGTCATCCTTGTAGTCCTTGTCGTCGTCATCCTTGTAGTCGGGCCCCTCGAGGATATCACCGGTTCTAGACATG) were annealed at 100°C for 1 min, slow-cooled to room temperature, and then digestion was performed on the annealing fragment, which has an XbaI site at its 5′-terminal and a BamHI site at its 3′-terminal, with XbaI and BamHI. Parent plasmid plenti-CMV-MCS-RFP-SV-PURO was digested with XbaI and BamHI to remove the DsRed2 gene. The digested annealed fragment was inserted into XbaI and BamHI sites in place of DsRed2 in plenti-CMV-MCS-RFP-SV-PURO to construct the plenti-CMV-MES-2ΧFLAG plasmid. This insert contains multiple enzyme sites of XbaI, Agel, EcoRV, Xhol, and Apal following the 2xFLAG-stop codon and BamHI site. We used plenti-CMV-MES-2XFLAG plasmid to construct plenti-CMV-VCAM1-2XFLAG. To construct the plenti-CMV-VCAM1-2XFLAG-puro plasmid, cDNA from KSHV-HUVEC mRNA was used as a DNA template with primer-F, GCATACCGGTATGCCTGGGAAGATGG (which has an AgeI site), and primer-R, GTATGGGCCCCACTTTTGATTTCTGTGCTTC (which has an ApaI site), for PCR to clone the VCAM1 gene. plenti-CMV-MES-2XFLAG plasmid and PCR product were subjected to digestion with AgeI and ApaI restrictive enzymes. Digested PCR products were inserted into AgeI and Apal sites of plenti-CMV-MES-2XFLAG plasmid to construct plenti-VCAM1-2XFLAG.

### Stable cells

Preparation of lentivirus was performed with the Viral Power kit (Invitrogen, cat. no. K497500) following the manufacturer’s instructions. Briefly, 70%–80% confluency of 293FT cells was placed into a 10-cm dish. Next, the media was replaced with 5 mL of medium without antibiotics. Nine micrograms of the ViraPower Packaging Mix and 3 μg of pLenti-CMV-MCS-vFLIP_2XFLAG_ or plenti-CMV-MES-2XFLAG were added into 1.5 mL of Opti-MEM I Medium. Thirty-six microliters of Lipofectamine 2000 was diluted in another 1.5 mL of Opti-MEM I Medium. The diluted plasmids and Lipofectamine 2000 were incubated for 5 min, respectively, and then combined and incubated at room temperature for 20 min. The mixture was added into the cells. Twenty-four hours post-transfection, the medium containing the DNA Lipofectamine 2000 complexes was removed and replaced with 10 mL of complete culture medium without antibiotics. Cells were incubated at 37°C, 5% CO_2_ for 72 h. Supernatant containing lentivirus was clarified by centrifugation and filtered through a 0.45-μm filter to remove cell debris. 1.2 × 10^5^ of hTERT-HUVEC were suspended in 1 mL of filtered supernatant containing lentivirus and were plated in one well of a 24-well plate. The cells were incubated at 37°C, 5% CO_2_ for 24 h. Old medium was replaced with 1 mL of fresh medium with a final concentration of 0.5 μg/mL of puromycin for stable cell selection. To test whether the stable cell line expressed vFLIP, lysates were evaluated by Western blot with anti-FLAG antibody.

The supernatant of lentivirus made from lentiCRISPR-sgVCAM1 and lentiCRISPR-V2 was added to hTERT-hygromycin HUVECs for 24 h, and then 10 μg/mL blasticidin was added for selection to obtain VCAM1 KO HUVEC and EV-HUVEC cell lines, respectively. Lentivirus produced from pLenti-CMV-VCAM1-2XFLAG and pLenti-CMV-mes-2XFLAG was used to transduce the VCAM1 KO HUVEC to build VCAM1 KO EV HUVEC and rVCAM1_FLAG_ HUVEC cell lines with 10 μg/mL blasticidin and 0.5 μg/mL puromycin for selection. All stable cell lines were tested by Western blot.

To prepare mutant vFLIP-KO KSHV HUVEC and wild-type vFLIP_FLAG_ KSHV HUVEC cell lines, 70% confluent iSLK.BAC16-vFLIP_FLAG_ or iSLK.BAC16-vFLIP KO, which were kindly provided by Dr. Jae U. Jung (48), were placed in one well of a 6-well plate for incubation at 37°C, 5% CO_2_ for 24 h. The next day, 2.5 mL of 2% FBS-DMEM containing 2 μg/mL of doxycycline and 1 mM of sodium butyrate were added and incubated at 37°C, 5% CO_2_ for 48 h for reactivation. Supernatant was collected and spun at 3,500 rpm for 20 min at 4°C. Collected supernatant was filtered through a 0.45-μm filter. Then, 1 mL of supernatant containing WT vFLIP_FLAG_ KSHV or vFLIP-deleted KSHV viruses was added into hTERT-neomycin-HUVEC cells in 70% confluency for infection at 37°C, 5% CO_2_ for 24 h. Infected HUVEC cells were selected with 100 μg/mL of hygromycin to establish stable WT vFLIP_FLAG_ KSHV-HUVEC or vFLIP-deleted KSHV-HUVEC cell lines.

### Adhesion assay

The adhesion assay was performed as previously described (49), but improved as follows. In brief, 200 µL of 0.1% gelatin was aliquoted into one well of a 48-well plate for coating for 1 h at 37°C and 5% CO_2_. After coating, 1 × 10^5^ endothelial cells were then seeded and allowed to form a monolayer in triplicate for each sample. Cells were then treated with PDTC or anti-VCAM1 antibody as described in figure legends. THP-1 cells were stained with 1 µL of stock solution of CellTrace Far Red Cell Proliferation Kit following the manufacturer’s instruction with modifications. THP-1 cells (1 × 10^6^/mL PBS) were stained with 1 μL of CellTrace Far Red staining solution stock in an incubator at 37°C, 5% CO_2_ for 20 min. Then, 5 mL of DMEM containing 1% BSA, 2 mM CaCl_2_, and 2 mM MgCl_2_ was added and incubated at 37°C, 5% CO_2_ for 5 min. Stained cells were centrifuged at 1,000 × *g* for 3 min and were suspended in 1 mL of DMEM containing 0.5% BSA, 2 mM CaCl_2_, and 2 mM MgCl_2_ for the adhesion assay. Stained THP-1 cells (2 × 10^5^) were added to the monolayer of endothelial cells per well and were incubated at 37°C and 5% CO_2_ for 6 min. Next, unadherent THP-1 cells were removed by washing with wash buffer (1× PBS, 0.1% BSA, 200 nM MgCl_2_, 150 µM CaCl_2_) four times, 200 µL of 1× PBS was added, and fluorescence intensity was measured via the Cy5 channel on a Clariostar Microplate Reader and images were taken with a fluorescence microscope (Leica Thunder Imager). All experimental conditions were performed at least three independent times. Finally, the cells were harvested for Western blot analysis.

### Transendothelial migration assay

Sterile 6.5 mm Transwells with 8.0-µm Pore Polyester Membrane Inserts were purchased from Corning (product no. 3464). The transendothelial migration assay was performed as previously described (69) but was modified as follows: 2 × 10^5^ HUVEC or KSHV-HUVEC were placed in one insert of a 6.5 mm Transwell in triplicate for each sample. The insert and well contained 100 µL and 500 µL of complete medium, respectively. The endothelial cells were allowed to form a layer. To confirm that there was no leak in the cell layer, trypan blue solution (Sigma T8154) was added into one well of the insert. If no diffusion of trypan blue was observed through the cell layer from the insert to the well, the cell layer was treated with PDTC or anti-VCAM1 antibody as described in the figure legends. Next, stained THP-1 cells were added into the insert. These cells were stained with CellTrace stock solution as described above in the Adhesion assay section. Stained THP-1 cells (2 × 10^5^) were added onto the endothelial layer in the insert. Then, the insert loaded with stained THP-1 cells on the endothelial cell layer was placed into a new well of a 24-well plate, which contained 500 µL of fresh complete medium with 50 ng/mL of MCP-1. Unless stated otherwise, the transwell was incubated at 37°C and 5% CO_2_ for 4 h for the THP-1 transendothelial migration assay. Stained THP-1 cells migrating through the endothelial cell layer down into the well were collected and placed into a 1.5-mL tube. Collected stained cells were washed with 1× PBS/0.1% BSA/200 nM MgCl_2_/150 µM CaCl_2_ four times. Finally, the stained cells in 100 µL of 1× PBS were placed in a well of a 96-well plate, fluorescence intensity was measured with a Clariostar Microplate Reader, and images were taken with a fluorescence microscope (Leica Thunder Imager). All experiments were performed with at least three independent biological replicates. Finally, the cells were harvested for Western blot analysis.

### Isolation of primary monocytes and B cells

Human monocytes and B cells were isolated from buffy coats (Gulf Coast Regional Blood Center) using the Monocyte Isolation Kit II (Miltenyi) and the B Cell Isolation Kit II (Miltenyi) according to the manufacturer’s instructions. Cells were stained with CD14 (61D3, eBiosciences) for monocyte isolation and with CD19 (LT19, Miltenyi) for B-cell isolation prior to analysis on a MACSQuant VYB cytometer (Miltenyi) and FlowJo software (Tree Star). Monocyte isolations containing >90% CD14+ cells and B-cell isolations containing ≥95% CD19+ cells by flow cytometer analysis were used.

### siRNA and reverse transfection

VCAM1 siRNA (small interfering RNA) (Guide strand 5′-UGCCCACAGUAAGGCAGGCUGUAAA-3′) and control siRNA (luciferase) (Guide strand 5′-CGUACGCGGAAUACUUCGATT-3′) (70) were purchased from Eurofins. siRNAs for knockdown of IKKα (Santa Cruz, sc-29365), RelB (Santa Cruz, 36402), NF-κB p52 (Santa Cruz; sc-29409), and NIK (Santa Cruz; sc-36065) were obtained from Santa Cruz Biotechnology, as well as an siRNA for NF-κB p65 knockdown (Cell Signaling; 6261). Reverse transfection of siRNAs was carried out using Lipofectamine RNAiMAX (Invitrogen) in Gibco Opti-MEM medium, at a final concentration of 50 nM of siRNA. Unless stated otherwise, cells were harvested 48 h post transfection for Western blot, adhesion assay, and at 72 h for transendothelial migration assays. Endothelial cells (2 × 10^6^) were placed in one well of a 6-well plate in reverse transfection for Western blot analysis, 2 × 10^5^ of the cells were placed into one well of a 48-well plate for the adhesion assay, and 3 × 10^5^ of the cells in one insert of a transwell were used for transendothelial migration assay for siRNA reverse transfection.

### Western blots and immunohistochemistry

A total of 5 × 10^5^ cells per well were seeded into one well of a 6-well plate and grown for 48 h up to 100% confluency. The cells were washed with plain EBM-2 medium twice. The cells were cultured in 2 mL of plain EBM-2, EBM-2 with 10% FBS, or EBM-2 plus 10% FBS containing the EBM-2 bullet supplement for 24 h, respectively. Then, the cells were scraped and lysed, and the lysate was mixed with 2× Laemmli buffer. Thirty-five micrograms of each protein sample was subjected to SDS-PAGE and transferred onto a nitrocellulose membrane. Western blot analysis was performed with the indicated antibodies.

For immunochemistry, slides of L1T2 xenograft tumor (42), which retain the complete KSHV episome, were graciously provided by Dr. Dittmer, and slides of human KS tumors were obtained from the AIDS and Cancer Specimen Resource (ACSR). Anti-VCAM1 antibody was obtained from Abcam (ab134047). Slides were deparaffinized using Histochoice clearing agent, and endogenous peroxidase activity was quenched with 3% H_2_O_2_. Antigens were exposed by heating sections for 25 min in Tris-EDTA buffer (10 mM Tris base, 1 mM EDTA solution, 0.05% Tween-20, pH 9.0) and cooled to room temperature. Nonspecific proteins were blocked using a blocking buffer (10% normal horse serum, 5% BSA, and 0.3% Triton X-100) for 30 min at room temperature, followed by an overnight incubation at 4°C in blocking buffer containing anti-VCAM1 antibody (1:500; Abcam; ab134047). The next day, slides were washed with 1× PBS, incubated with anti-rabbit secondary antibody for 50 min, followed by 30 min incubation in preformed Avidin-Biotin Complex (ABC) (Vectastain ABC kit; Vector Laboratories), after which slides were stained with Vector peroxidase substrate and washed. Slides were counterstained with hematoxylin QS (Vector Laboratories), dehydrated using graded alcohols and Histochoice, and mounted using Cytoseal XYL (Thermo Scientific). Dried slides were imaged using a Leica DM LA histology microscope.

## Data Availability

Western blot raw data are provided as a supplemental file.

## SUPPLEMENTAL MATERIAL

Supplemental Figures mbio.01499-26-s0001.pdf Figures S1 to S6.

Data set S1 mbio.01499-26-s0002.pdf Raw data for figures.

ASM does not own the copyrights to Supplemental Material that may be linked to, or accessed through, an article. The authors have granted ASM a non-exclusive, world-wide license to publish the Supplemental Material files. Please contact the corresponding author directly for reuse.
