# Supplementary material for: KSHV promotes leukocyte adhesion and transendothelial migration via induction of VCAM1
Source: mBio. 2026 Sep 8;17(10):e01499-26. doi: 10.1128/mbio.01499-26 (PMC13712882; doi:10.1128/mbio.01499-26)
Supplement: Supplementary file 1 [file mbio.01499-26-s0001.pdf]

Zhang et al. Figure S1

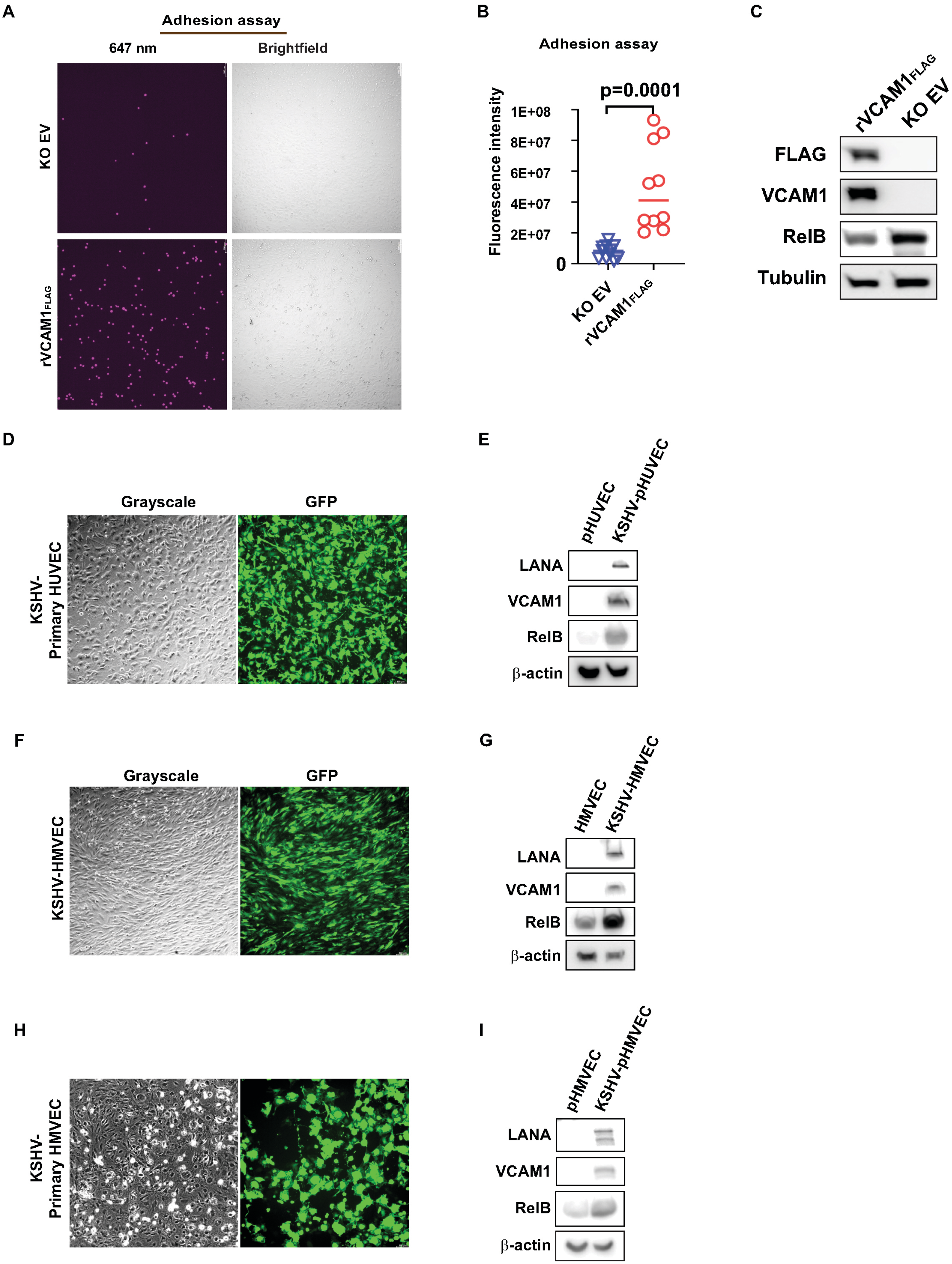

Zhang et al. Figure S2

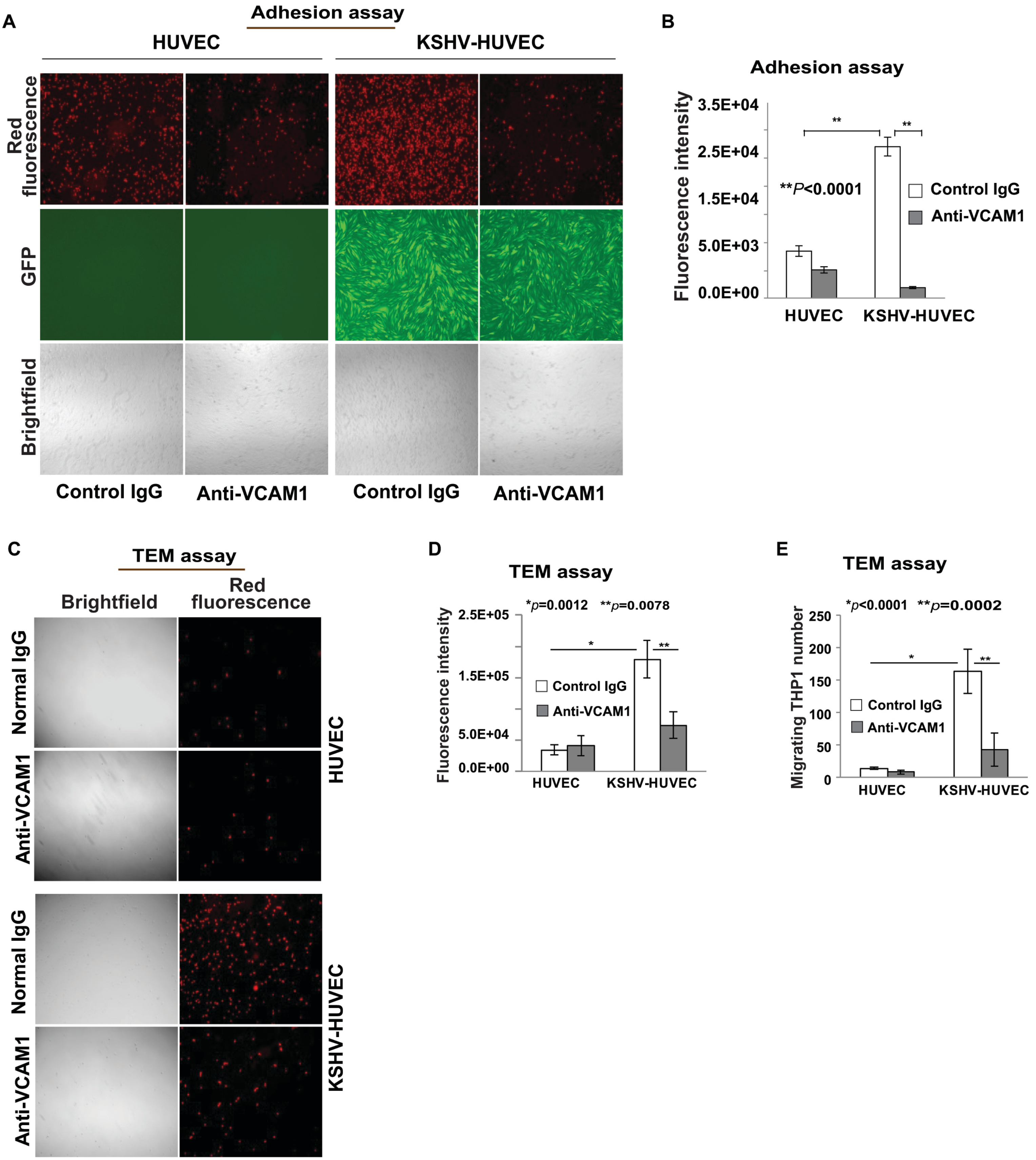

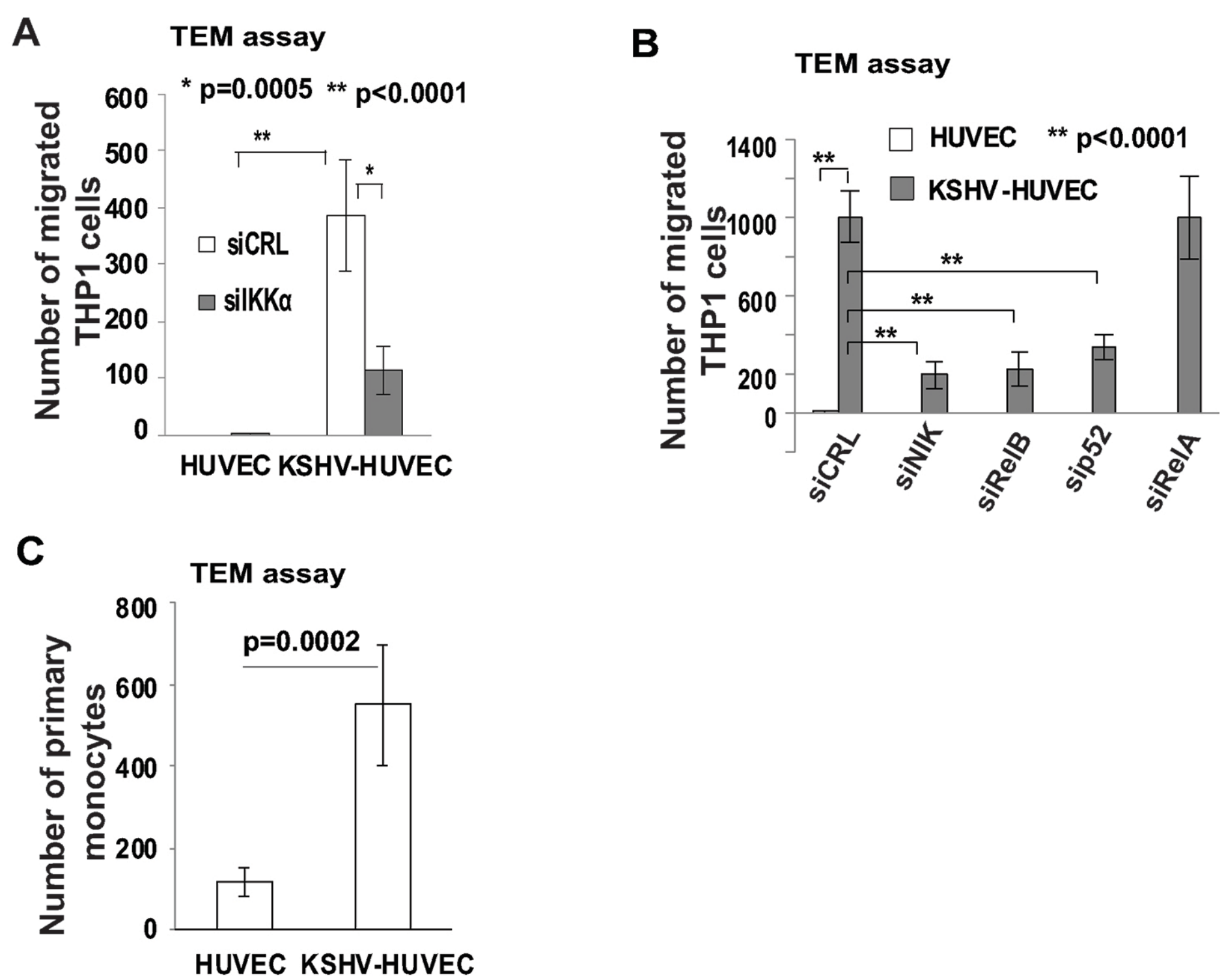
A
TEM assay
* p=0.0005 ** p<0.0001
Number of migrated THP1 cells
600
500
400
300
200
100
0
siCRL
siIKKα
**
*
HUVEC KSHV-HUVEC
B
TEM assay
HUVEC ** p<0.0001
KSHV-HUVEC
Number of migrated THP1 cells
1400
1000
600
200
0
siCRL
siNIK
siRelB
sip52
siRelA
C
TEM assay
Number of primary monocytes
800
600
400
200
0
p=0.0002
HUVEC KSHV-HUVEC

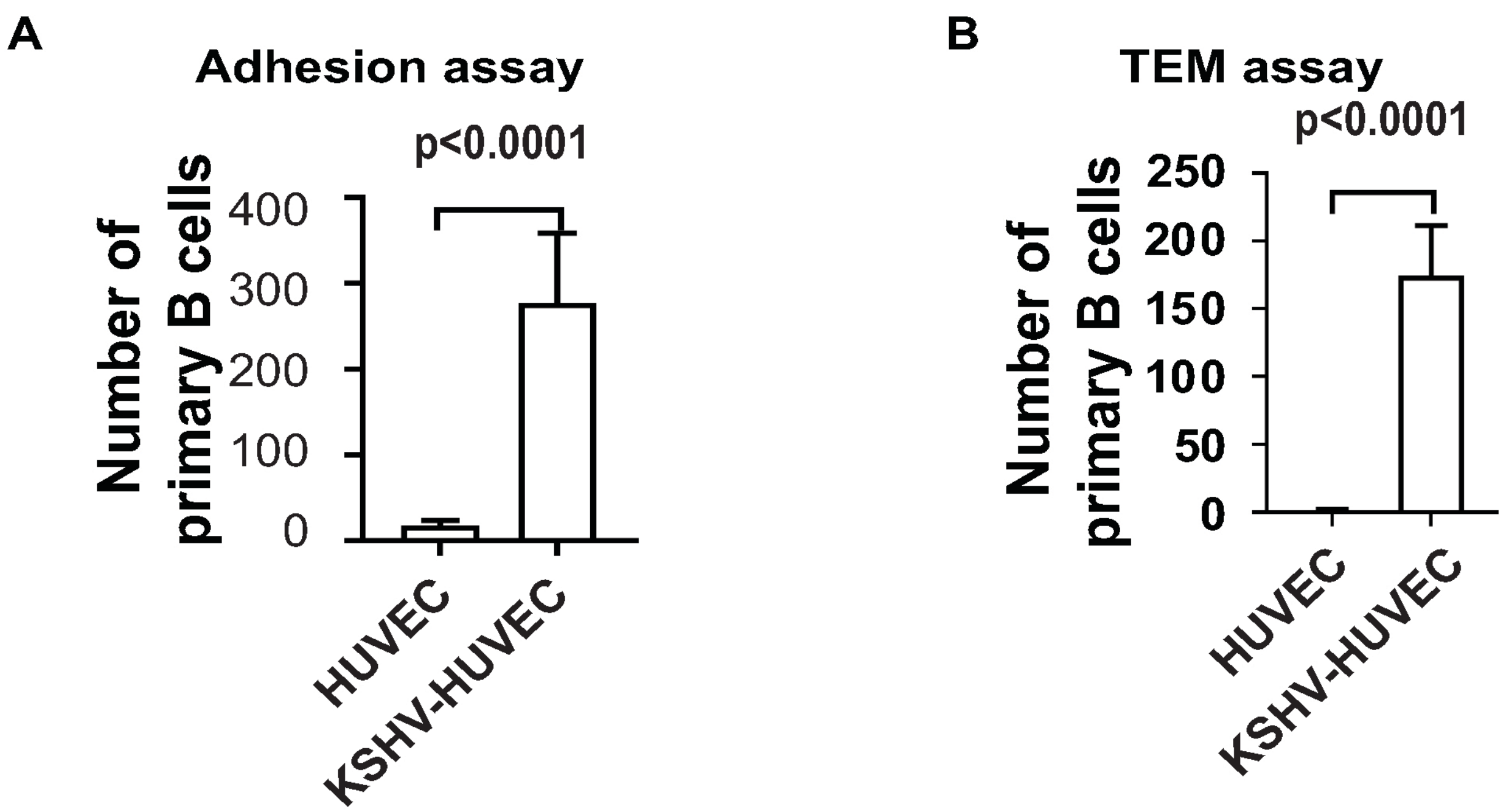
A
Adhesion assay
p<0.0001
Number of primary B cells
400
300
200
100
0
HUVEC
KSHV-HUVEC
B
TEM assay
p<0.0001
Number of primary B cells
250
200
150
100
50
0
HUVEC
KSHV-HUVEC

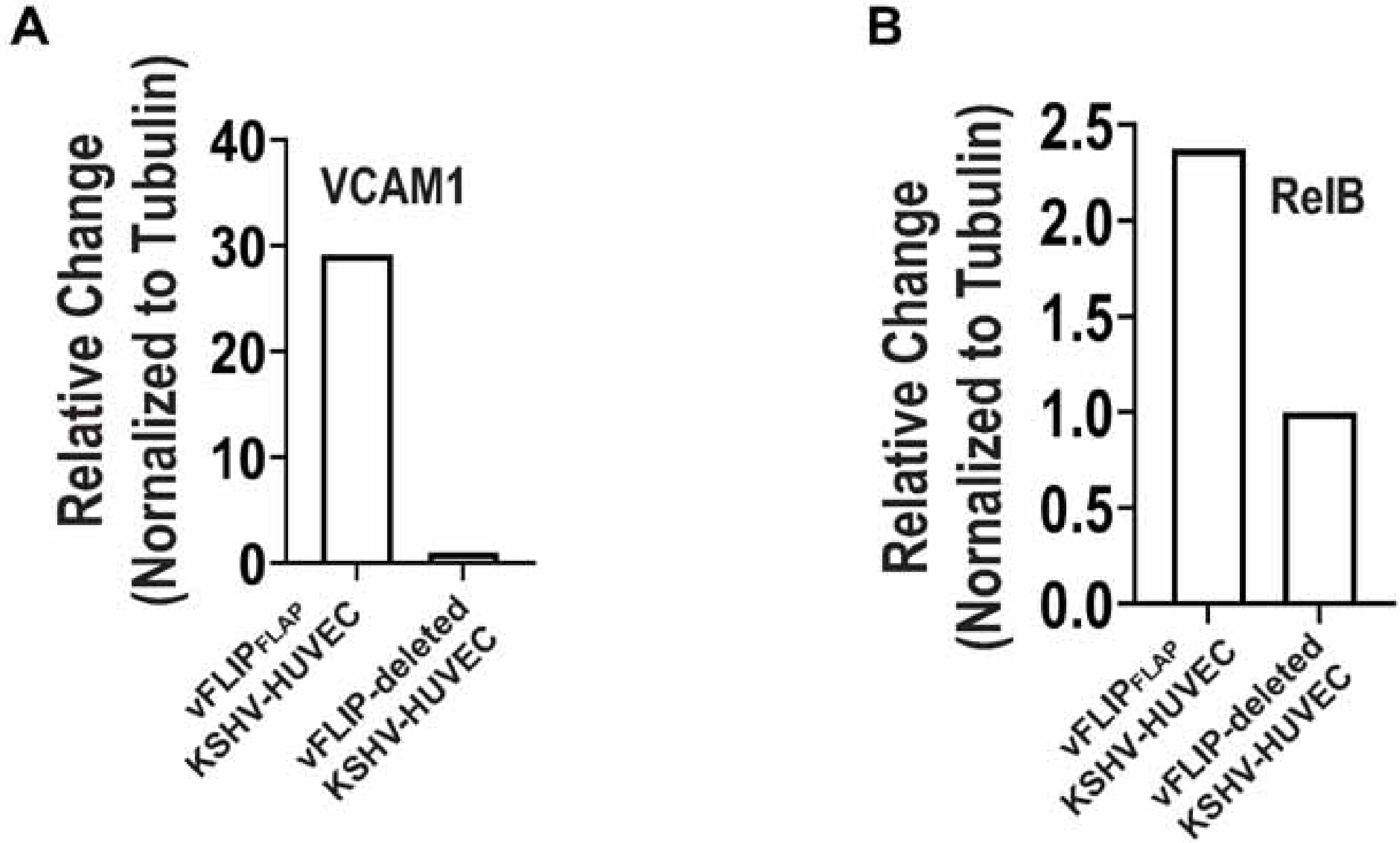
A
Relative Change
(Nornalized to Tubulin)
40
30
20
10
0
VCAM1
vFLIPFLAP
KSHV-HUVEC
vFLIP-deleted
KSHV-HUVEC
B
Relative Change
(Nornalized to Tubulin)
2.5
2.0
1.5
1.0
0.5
0.0
RelB
vFLIPFLAP
KSHV-HUVEC
vFLIP-deleted
KSHV-HUVEC

Zhang et al, Figure S6

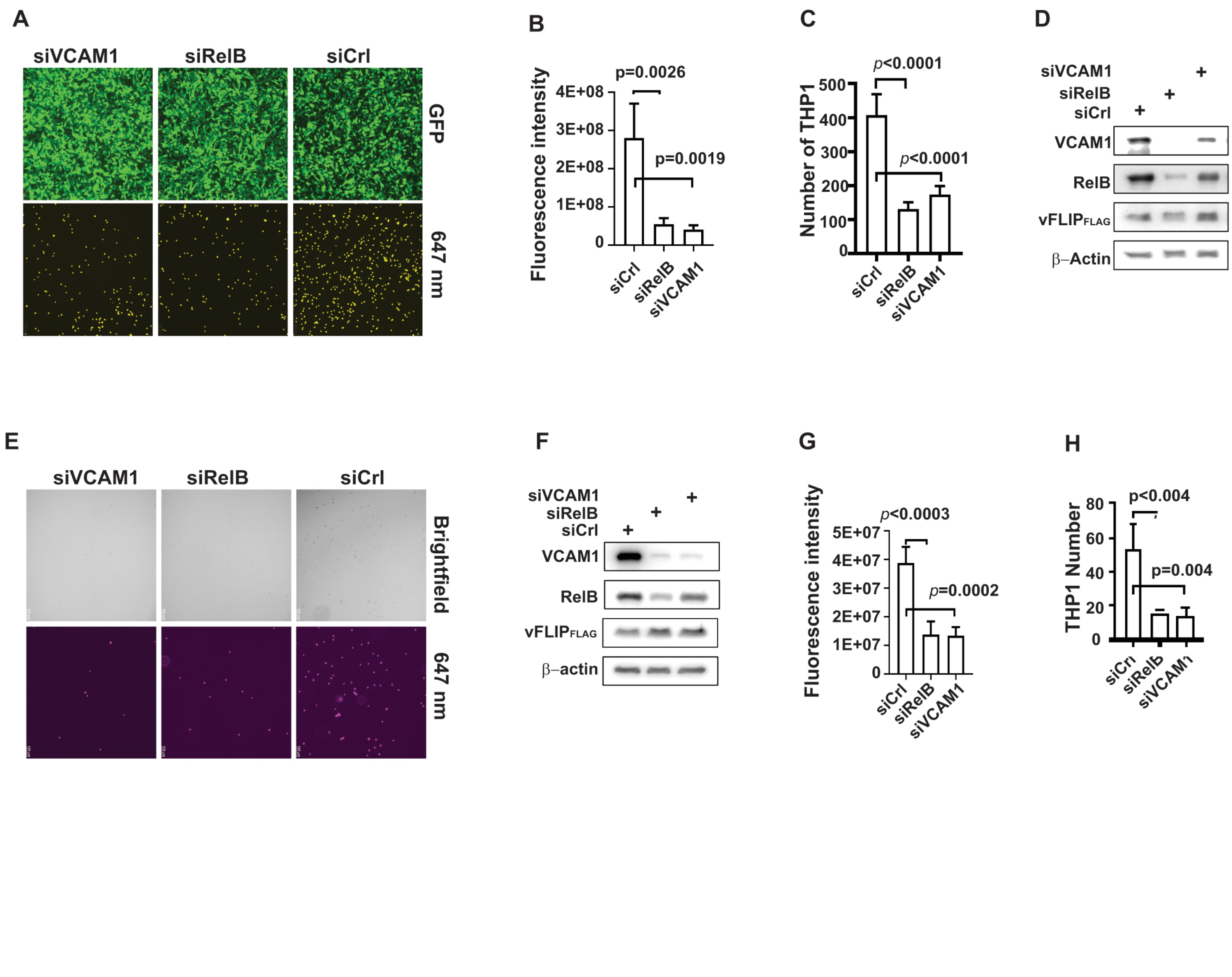

Supplemental Figure Legends

Fig. S1: VCAM1 promotes THP-1 adhesion

VCAM1 KO EV HUVEC and rVCAM1$_{FLAG}$ HUVEC cell lines were made by infecting VCAM1 KO HUVEC using empty vector lentivirus or VCAM1$_{FLAG}$ expressing lentivirus, followed by selection with 10 μg/ml blasticidin and 0.5 μg/ml puromycin. **(A)** Stained THP-1 cells were added to the rVCAM1$_{FLAG}$ HUVEC (rVCAM1 HUVEC) or VCAM1 KO EV HUVEC layer for adhesion assay, and images were taken at 10X magnification with a fluorescence microscope. **(B)** The fluorescence intensity of stained THP-1 cells adhered to the rVCAM1-Flag HUVEC or VCAM1 KO EV HUVEC layers was measured. **(C)** Lysates of rVCAM1$_{FLAG}$ HUVEC and VCAM1 KO EV HUVEC cells were subjected to Western blot analysis. The membrane was first probed with an anti-VCAM1 antibody, and then the blot was stripped and probed with anti-RelB, anti-FLAG (HRP) and anti-tubulin (HRP) antibodies, respectively. **(D)** Primary HUVEC cells were infected with KSHV virus for 5 days and images were taken under a fluorescence microscope at 10x magnification. **(E)** Western blot analysis was performed on lysates of de novo KSHV infected primary HUVECs. The membrane was first probed with an anti-VCAM1 antibody, and then the blot was stripped and probed with anti-LANA, anti-RelB and anti-β-actin, respectively. **(F)** Images of KSHV-infected HMVEC were taken at 10 x magnification with a fluorescence microscope. **(G)** Lysates of KSHV-HMVEC were subjected to Western blot. The membrane was first probed with an anti-VCAM1 antibody, and then the blot was stripped and probed with anti-LANA, anti-RelB and anti-β-actin, respectively. **(H)** Primary HMVEC were infected with KSHV virus for 5 days and then images of KSHV infected HMVEC were taken with a fluorescence microscope. **(I)** Lysates of de novo KSHV infected HMVEC were subjected to Western blot analysis. The membrane was first probed with an anti-VCAM1 antibody, and then the blot was stripped and probed with anti-LANA, anti-RelB and anti-β-actin, respectively. p-value of panel B

is the result of two-tailed Student's t-tests. The error bars represent the standard error of the means of ten independent biological replicates (n=10).

Fig. S2. Anti-VCAM1 antibody blocks THP-1 adhesion and TEM

An adhesion assay was performed. A layer of KSHV-HUVEC or HUVEC cells in one well of a 48-well plate was treated with 3 μg of anti-VCAM1 antibody in 50 μl of DMEM containing 0.5% BSA, 2 mM $CaCl_2$, and 2 mM $MgCl_2$ at room temperature for 10 min. Next, 200 μl of stained THP-1 (2 x 10^5) cells were added for 5 min. Cells were treated with 3 μg of normal IgG as control. **(A)** Images of the stained THP-1 cells were taken with 4X magnification using a fluorescence microscope. **(B)** Intensity of the stained THP-1 cells was measured using a Clariostar Microplate Reader. **(C)** A TEM assay was performed. The endothelial cell layer was treated with 3 μg of anti-VCAM1 antibody or normal IgG in 30 μl of DMEM containing 0.5% BSA, 2 mM $CaCl_2$, and 2 mM $MgCl_2$ in the incubator at 37°C, 5% CO_2 for 15 min with shaking every 3 minutes. Next, stained THP-1 cells were added and incubated for 2.5 h. Stained THP-1 cells that had migrated through the endothelial cell layer were imaged at 4X magnification using a fluorescence microscope. **(D)** The intensity of the stained THP-1 cells that had migrated through the endothelial cell layer was measured with a Clariostar Microplate reader. **(E)** The number of the stained THP-1 cells that had migrated through the endothelial cell layer was counted using a fluorescence microscope. For panel B and D-E, p-values are the result of two-tailed Student's t-tests; exact p-values are indicated directly within each corresponding figure panel. The error bars represent the standard error of the means of three independent biological replicates (n=3) for each corresponding figure panel.

Fig. S3. **TEM assay of leukocytes**. (**A** and **B**) The number of stained THP-1 cells that had migrated through the endothelial cell layer treated with siIKKα, siNIK, sip52, siRelB, and siRelA in the TEM assays described in Fig. 4 was counted under a microscope. **(C)** The number of stained primary monocytes that migrated across the endothelial cell layers in the TEM assay is

shown. All p-values of panel A-C are the result of two-tailed Student's t-tests; exact p-values are indicated directly within each corresponding figure panel. The error bars represent the standard error of the means of three independent biological replicates (n=3) for each corresponding panel.

Fig. S4. **Adhesion and TEM assays of primary B cells. (A)** The number of stained primary B cells that adhered to the endothelial cell layers in the adhesion assay in Fig. 4 were counted with a fluorescence microscope. **(B)** The number of stained primary B cells that migrated through the endothelial cell layers in the TEM assay in Fig. 4 were counted with a fluorescence microscope. p-values of panel A-B are results of two-tailed Student's t-tests; exact p-values are indicated directly within each corresponding figure panel. The error bars represent the standard error of the means of three independent biological replicates (n=3) for each panel A and B.

Fig. S5. **KSHV vFLIP contributes to TEM via expression of VCAM1 and RelB. (A)** Comparison of the fold change of VCAM1 expression in WT $vFLIP_{FLAG}$ KSHV-HUVEC and vFLIP-deleted KSHV-HUVEC cells. The band density was quantified using the BIORAD ChemiDoc Imaging System. **(B)** Comparison of the fold change of RelB expression in WT $vFLIP_{FLAG}$ KSHV-HUVEC and vFLIP-deleted KSHV-HUVEC cells. The band density was quantified using the BIORAD ChemiDoc Imaging System.

Fig. S6. VCAM1 and RelB aid TEM of THP-1 cells. 2 X 10^5 of WT $vFLIP_{FLAG}$ KSHV- HUVEC cells were plated in one well of a 48 well plate for an adhesion assay. Stained THP-1 cells were added to the layer of WT $vFLIP_{FLAG}$ KSHV-HUVEC cells that had been treated with siCrl, siRelB and siVCAM1. (**A**) Images of stained THP-1 cells adhered to the endothelial cell layer were taken at 10X magnification with a fluorescent microscope**.** (**B**) Fluorescence intensity and **(C)** the number of stained THP-1 cells was measured and counted under a fluorescent microscope. (**D**) Western blot analysis confirmed the decrease of RelB and VCAM1 in siRelB and siVCAM1-treated WT $vFLIP_{FLAG}$ KSHV-HUVEC compared to siCrl-treated cells in adhesion assay. The membrane was first probed with an anti-VCAM1 antibody, and then the blot was stripped and

probed with anti-RelB, anti-FLAG (HRP) and anti-β-actin antibodies, respectively. (**E**) 3 X 10^5 of WT $vFLIP_{FLAG}$ KSHV-HUVEC cells were plated in one insert for reverse transfection. 2 X 10^5 of stained THP-1 were added to the layer of WT $vFLIP_{FLAG}$ KSHV-HUVEC that had been treated with siCrl, siRelB and siVCAM1 for a TEM assay. Images of the stained THP-1 cells that had migrated through the WT $vFLIP_{FLAG}$ KSHV-infected endothelial cell layer treated with siCrl, siRelB, or siVCAM1 were taken with a fluorescence microscope. **(F)** Knockdown of RelB and VCAM1 in the TEM assay was confirmed by Western blot analysis. The membrane was first probed with an anti-VCAM1 antibody, and then the blot was stripped and probed with anti-RelB, anti-FLAG (HRP) and anti-β-actin antibodies, respectively. **(G)** Fluorescence intensity and **(H)** the number of stained THP-1 cells migrating through the WT $vFLIP_{FLAG}$ KSHV-infected endothelial cell layer treated with siCrl, siRelB, or siVCAM1 was measured and counted using a fluorescence microscope. For panel BC and GH, all p-values are the result of two-tailed Student's t-tests; exact p-values are indicated directly within each corresponding figure panel. The error bars represent the standard error of the means of three independent biological replicates (n=3) for each corresponding panel.
