# Supplementary material for: KSHV promotes leukocyte adhesion and transendothelial migration via induction of VCAM1
Source: mBio. 2026 Sep 8;17(10):e01499-26. doi: 10.1128/mbio.01499-26 (PMC13712882; doi:10.1128/mbio.01499-26)
Supplement: Supplementary file 2 [file mbio.01499-26-s0002.pdf]

Fig. 1A

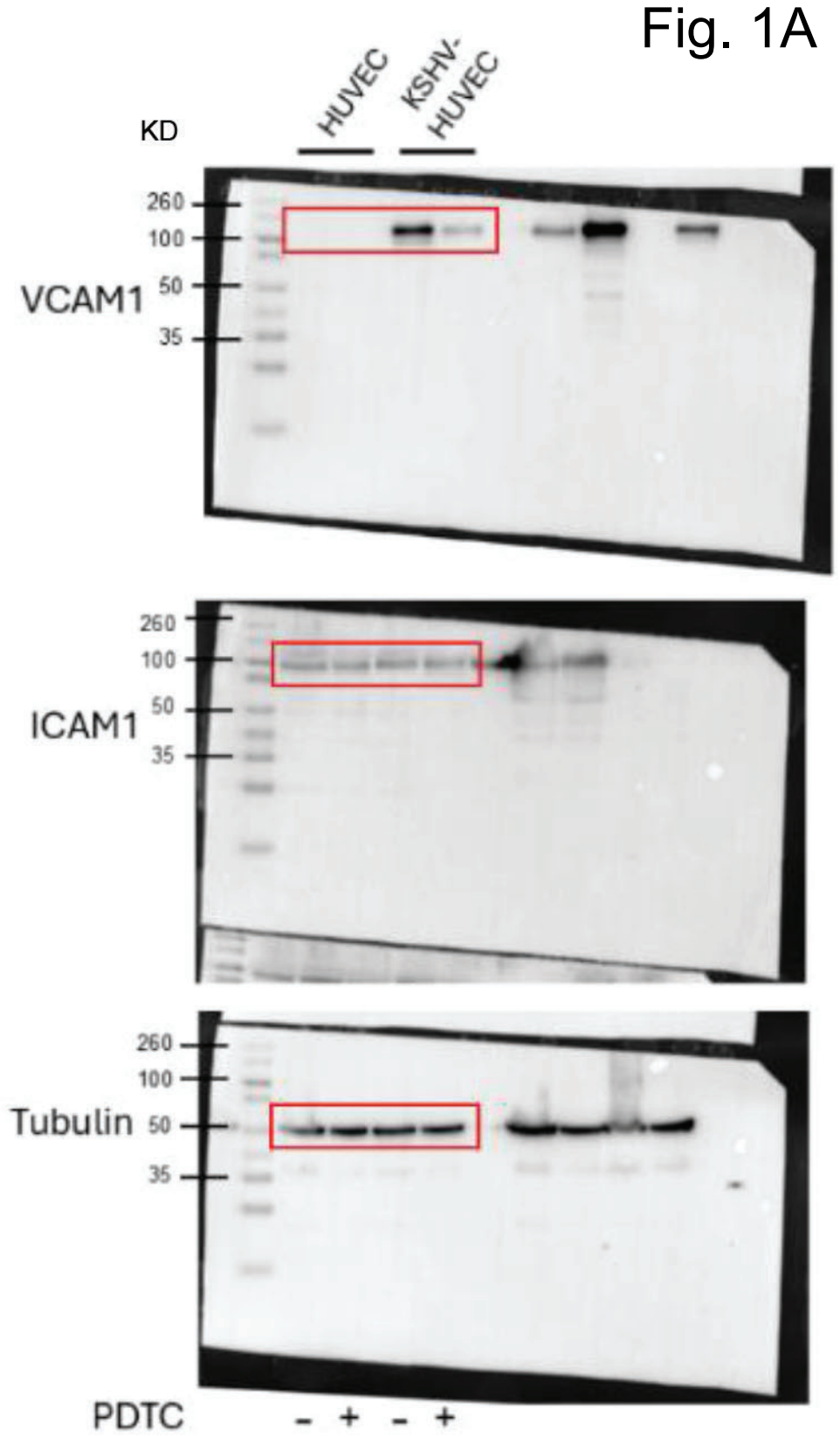

Fig. 1C

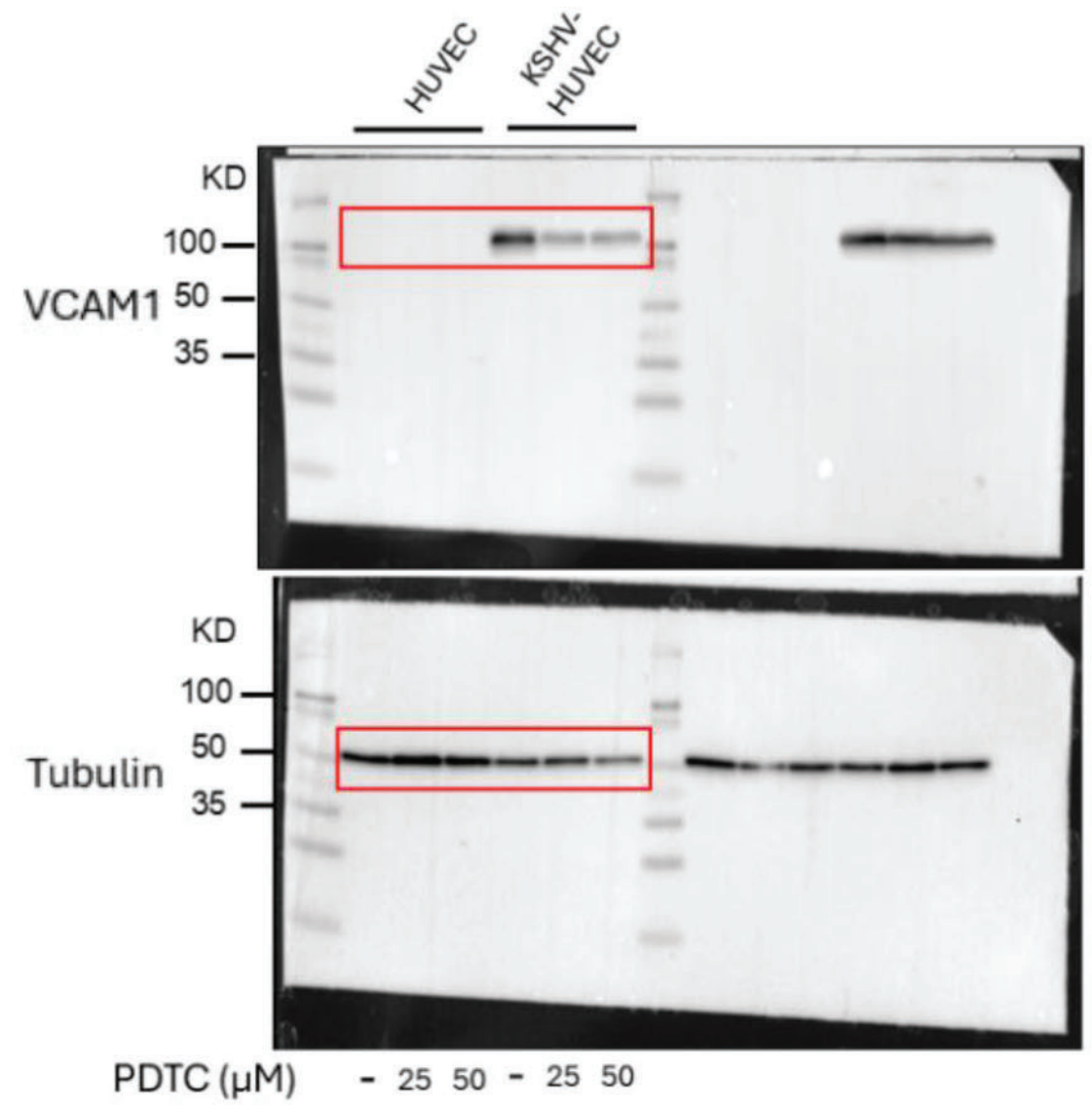

Fig. 1F

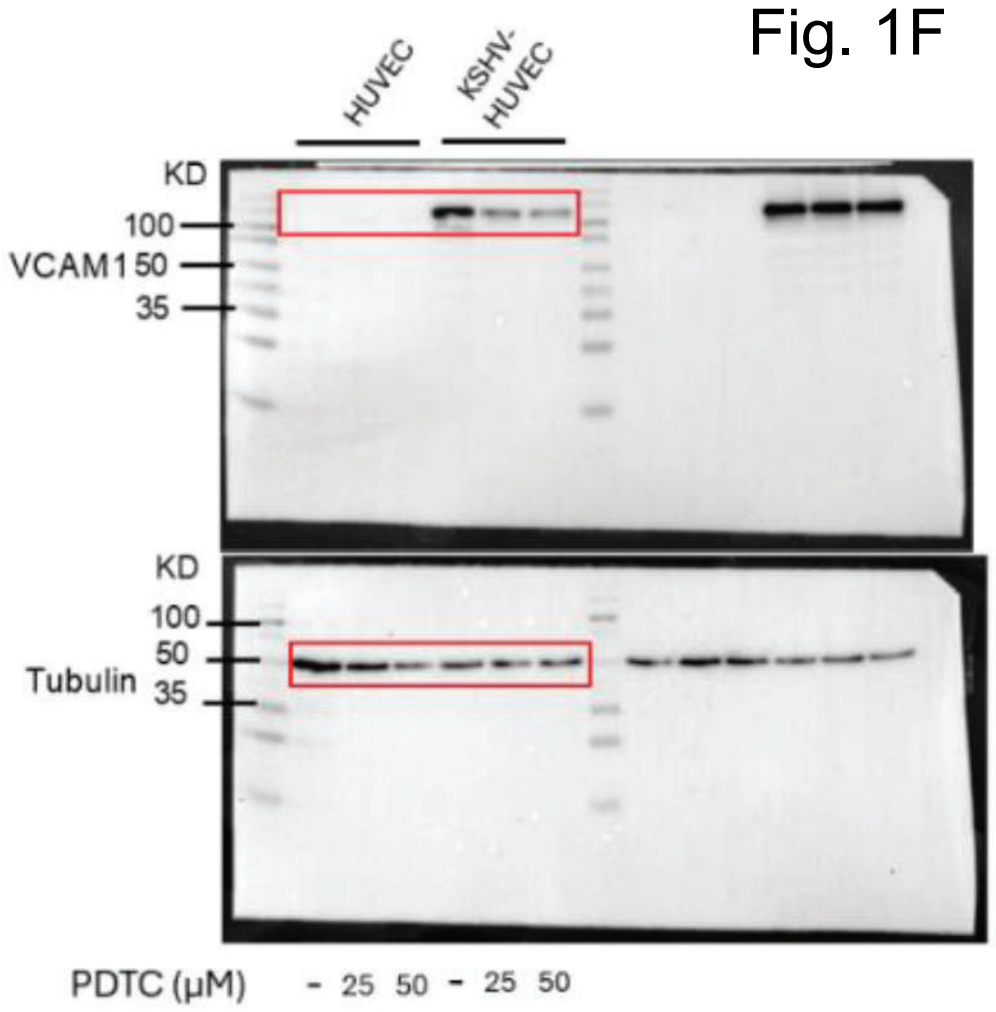

Fig. 1J

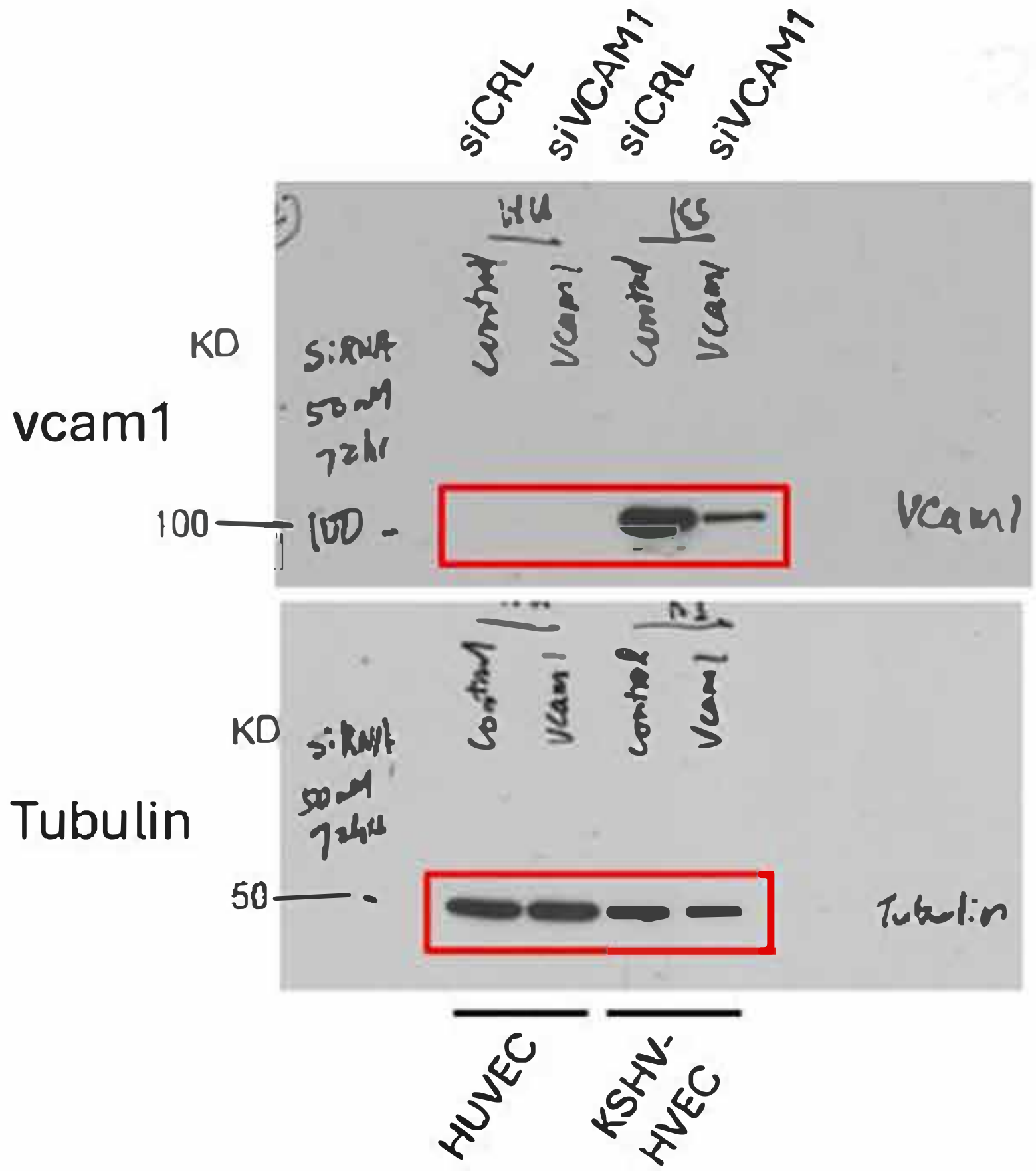
siCRL
siVCAM1
siCRL
siVCAM1
KD
vcam1
100
Tubulin
50
HUVEC
KSHV-HVEC

Fig. 1M

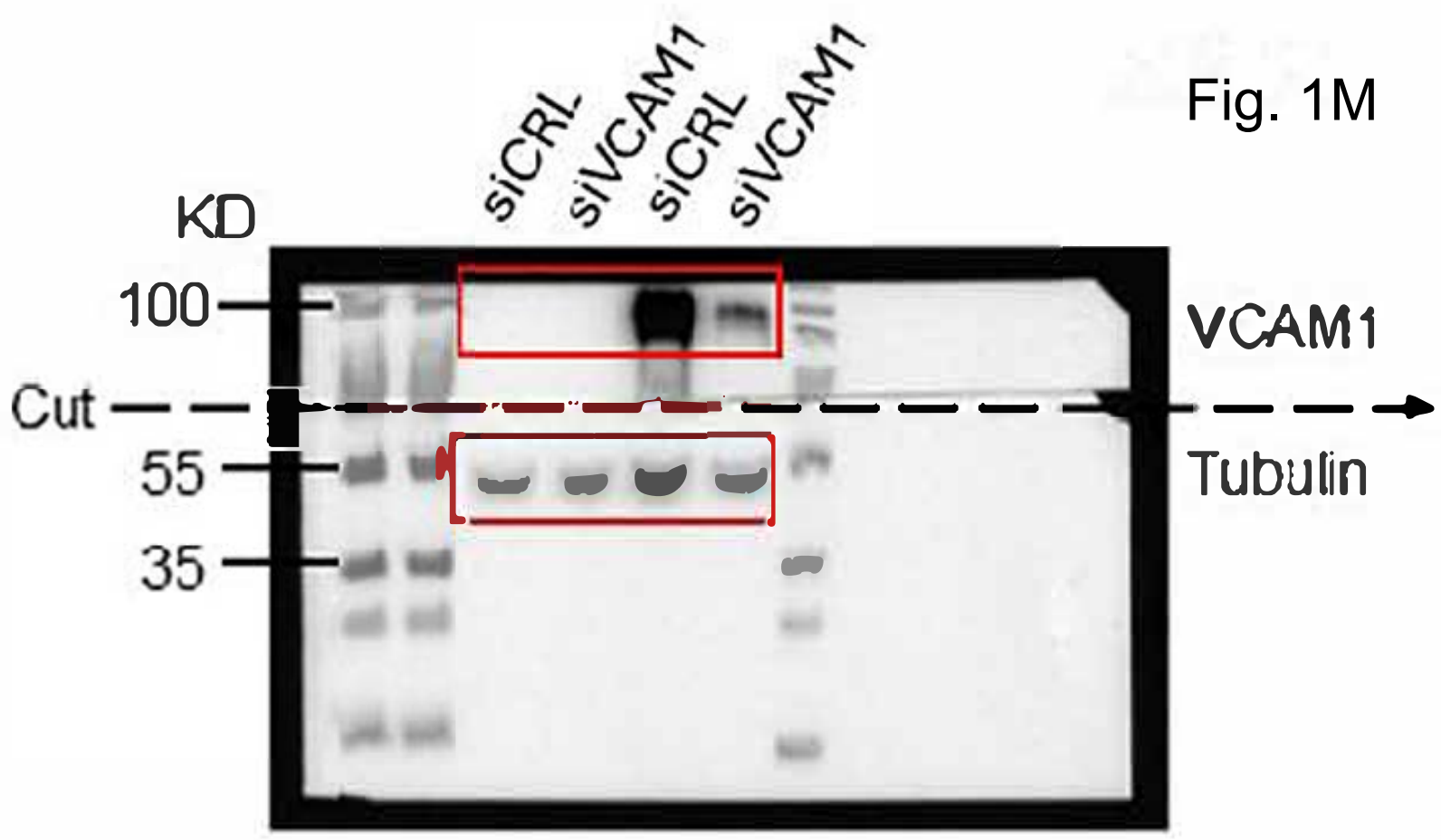
siCRL
siVCAM1
siCRL
siVCAM1
KD
100
Cut
55
35
VCAM1
Tubulin

Fig. 2A

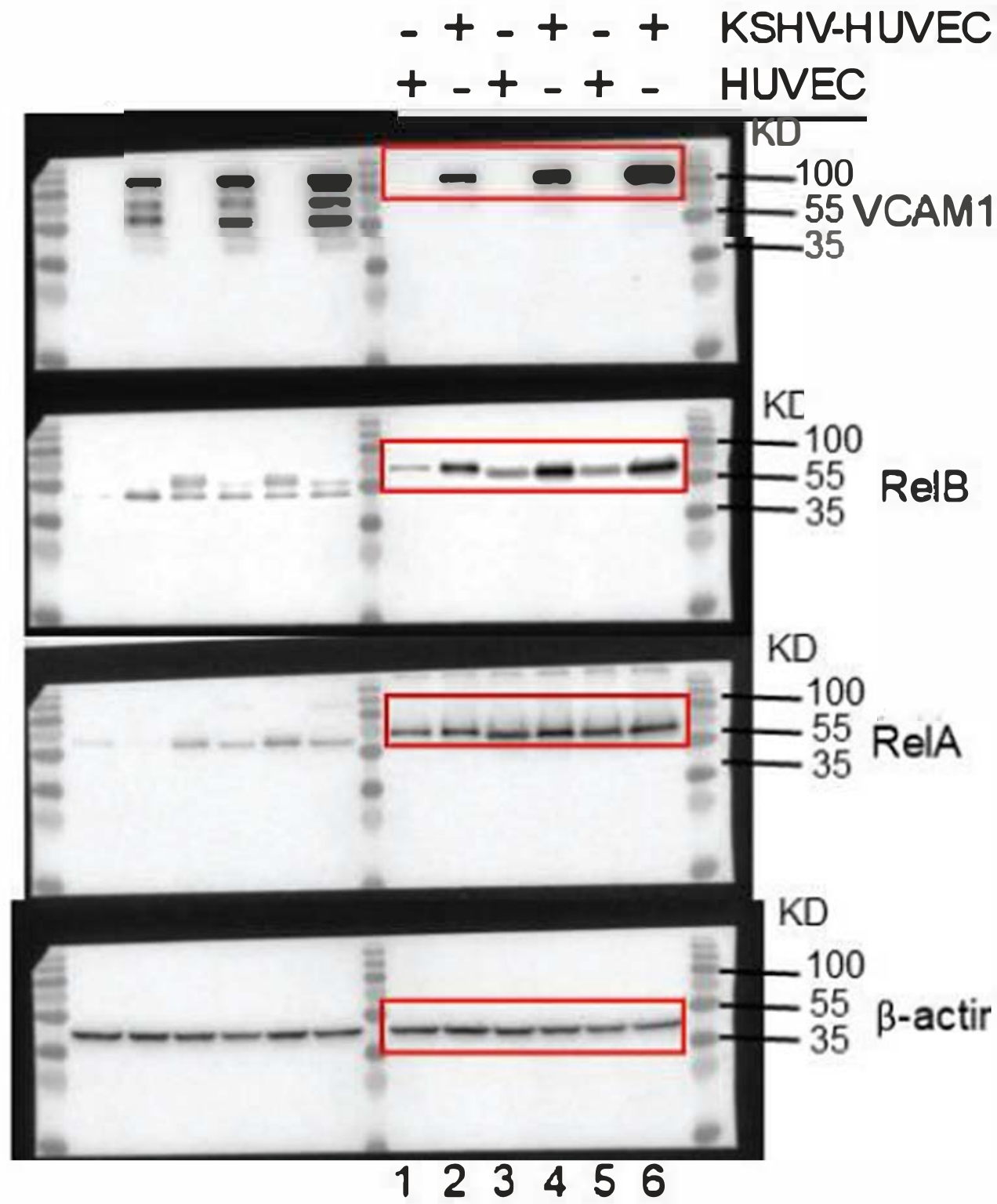

- + - + - + KSHV-HUVEC
+ - + - + - HUVEC
KD
100
55
35
VCAM1
RelB
RelA
β-actir
1 2 3 4 5 6

Fig. 2B

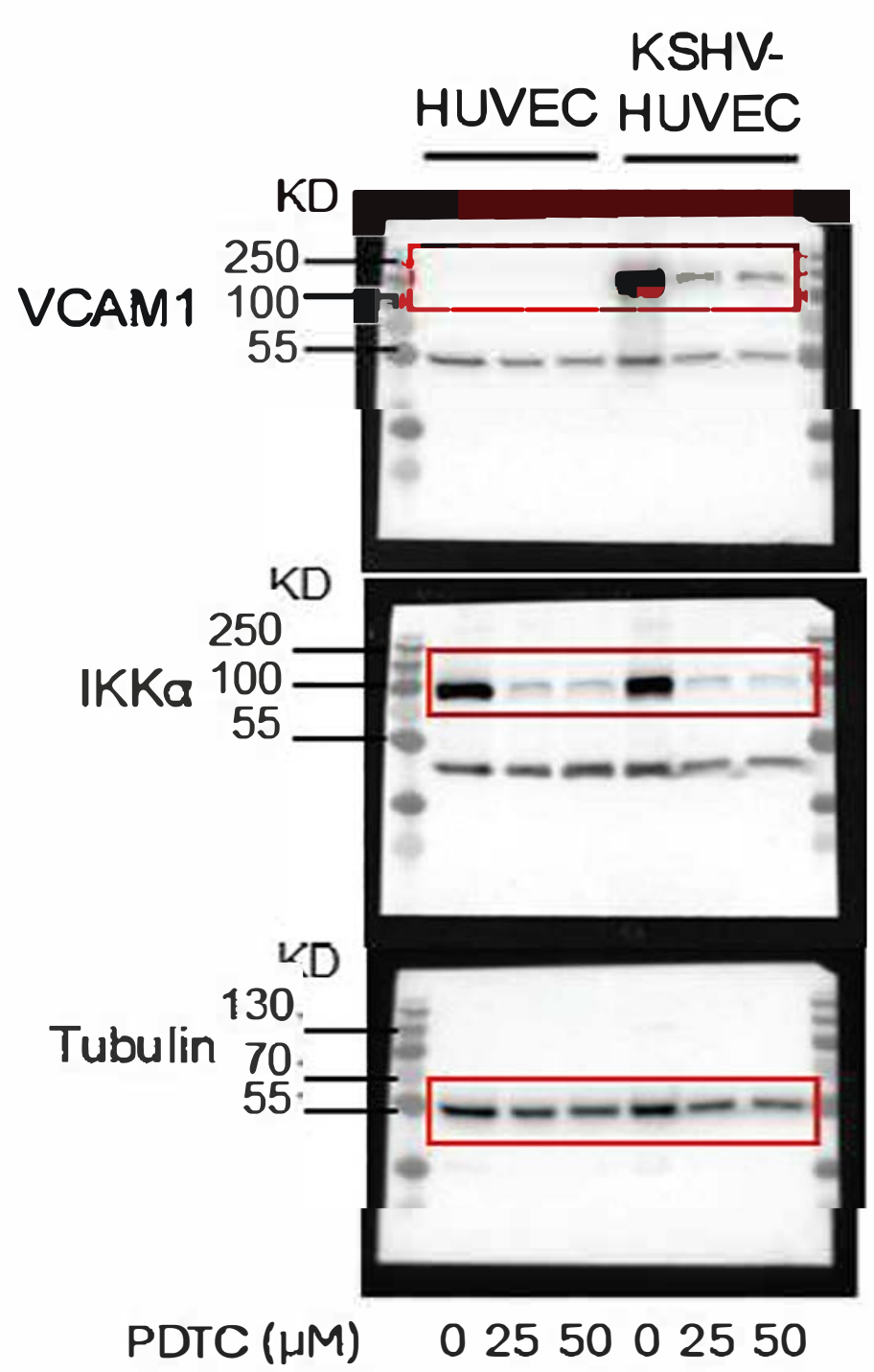

HUVEC
KSHV-HUVEC
KD
250
100
55
VCAM1
IKKα
130
70
Tubulin
PDTC (μM)
0 25 50 0 25 50

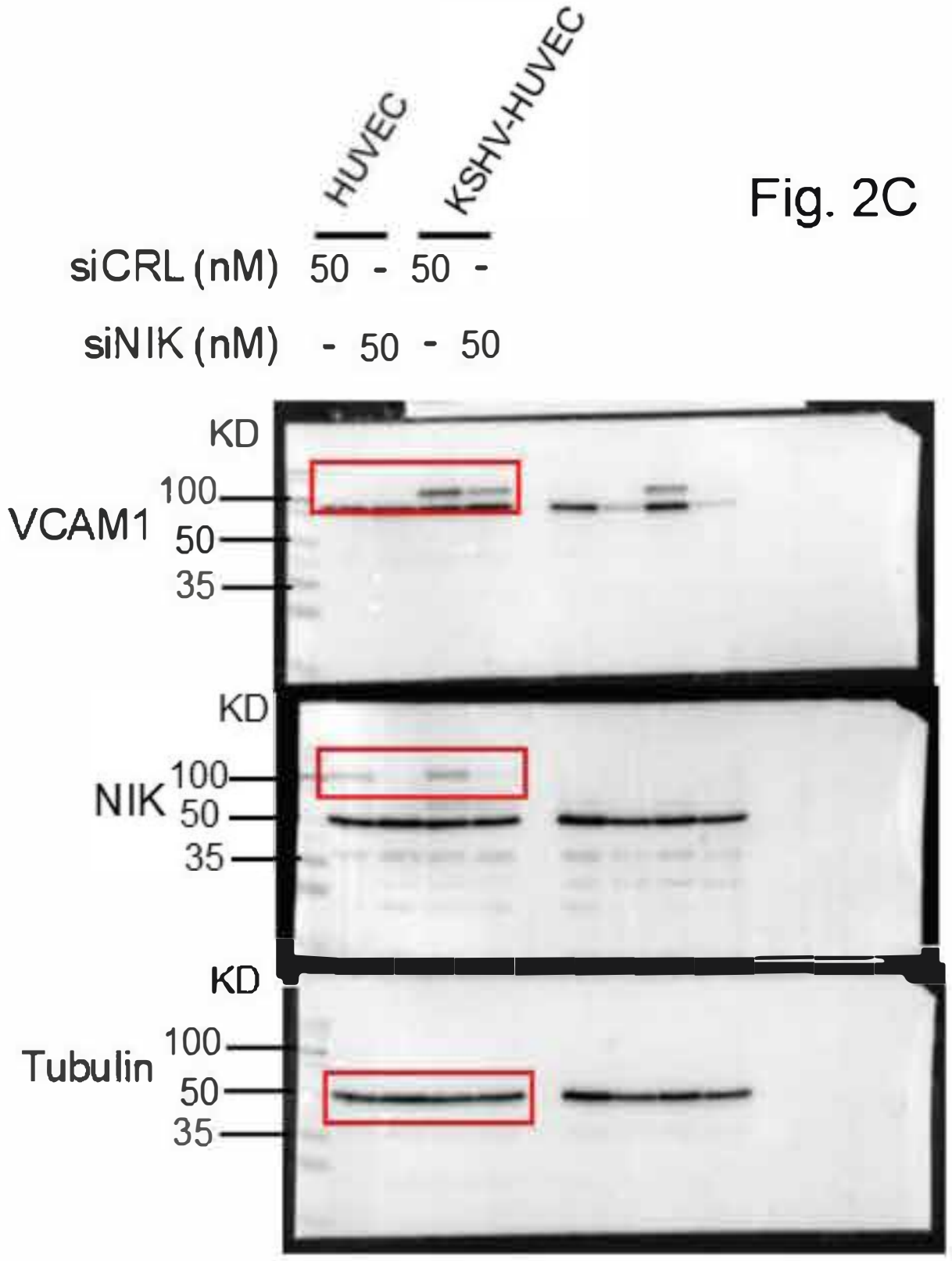
HUVEC
KSHV-HUVEC
siCRL (nM) 50 - 50 -
siNIK (nM) - 50 - 50
KD
100
50
35
VCAM1
NIK
Tubulin

Fig. 2C

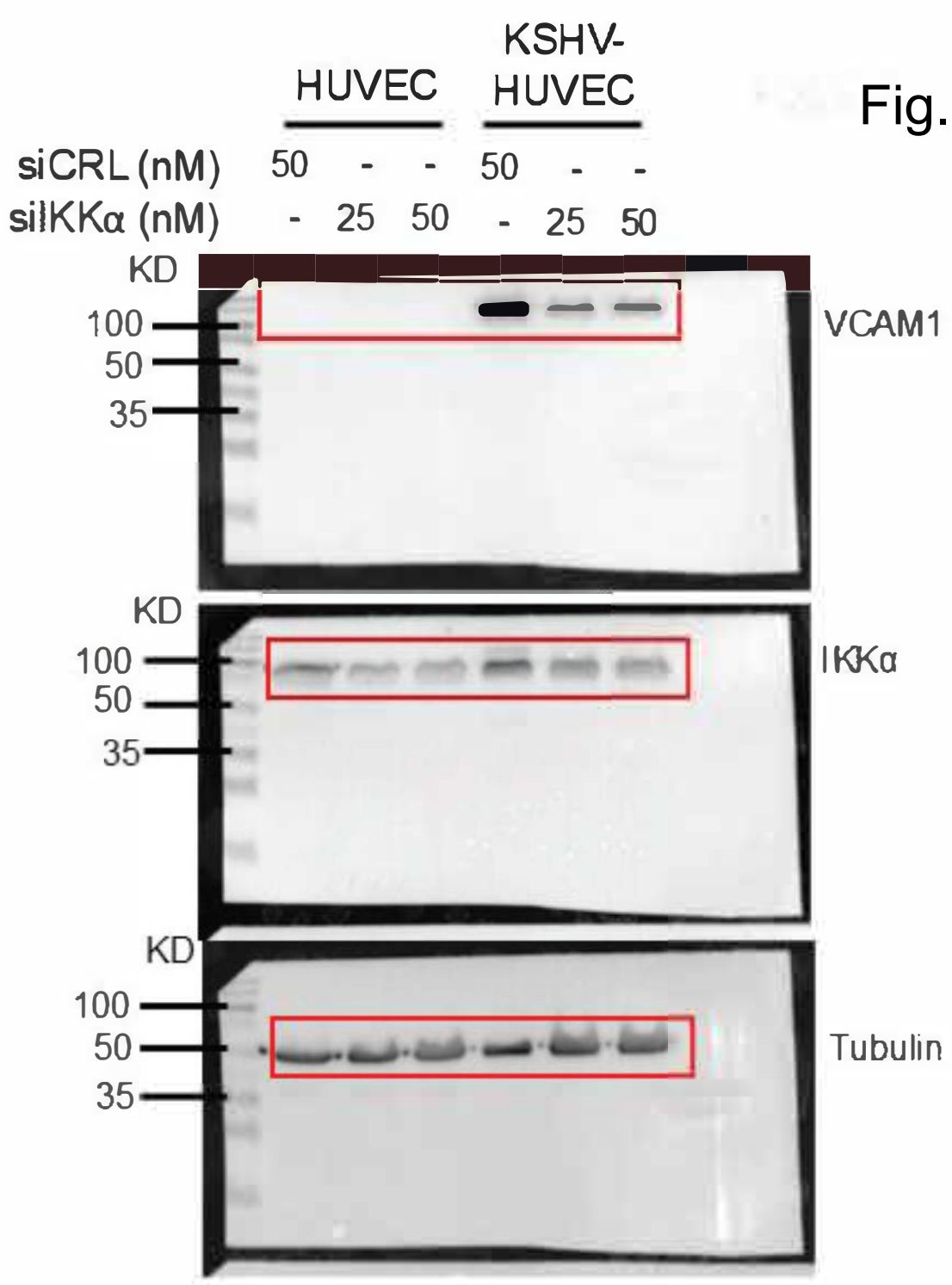
HUVEC
KSHV-HUVEC
siCRL (nM) 50 - - 50 - -
siIKKα (nM) - 25 50 - 25 50
KD
100
50
35
VCAM1
IKKα
Tubulin

Fig. 2D

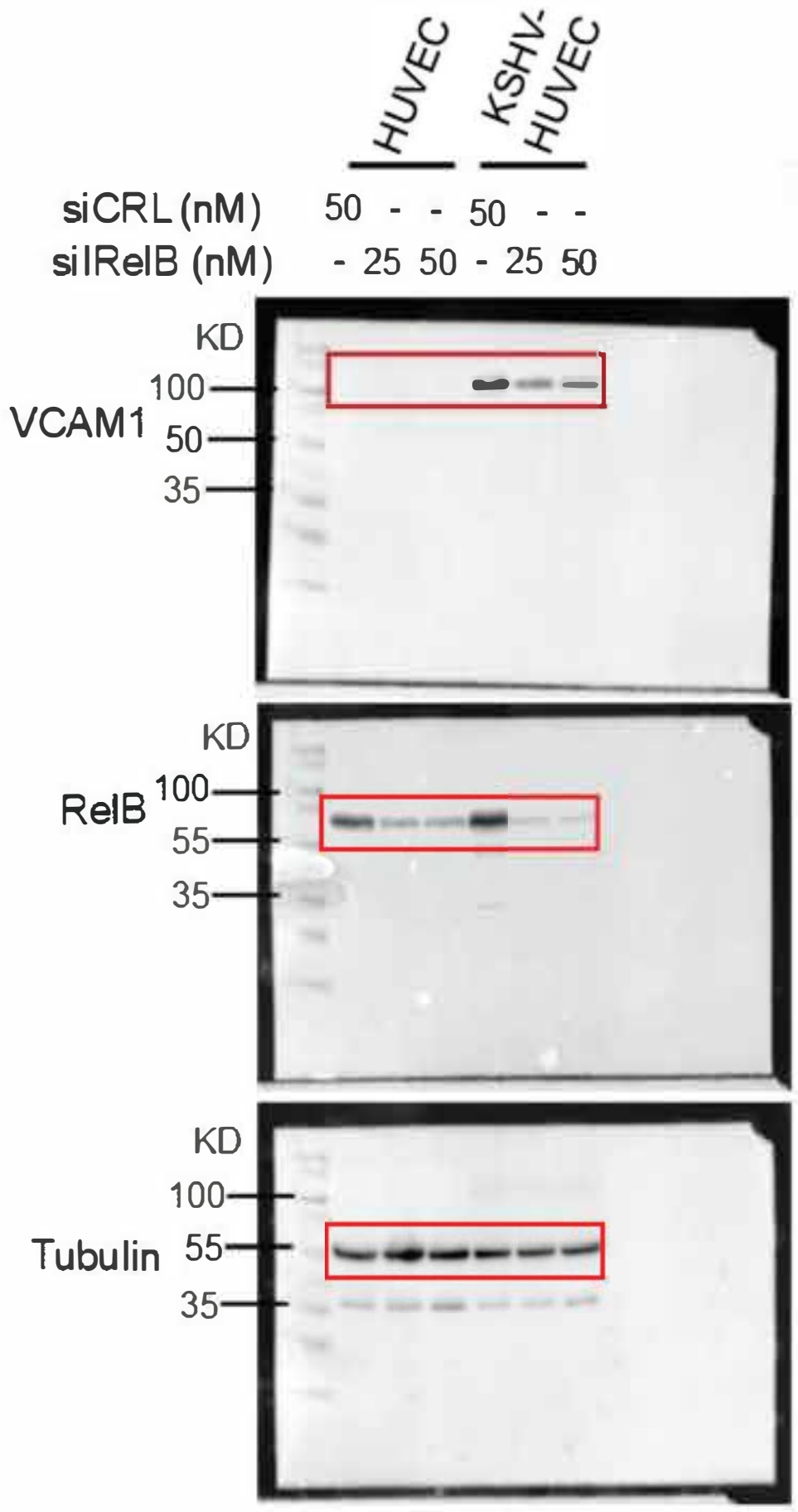
HUVEC
KSHV-HUVEC
siCRL (nM) 50 - - 50 - -
siIRelB (nM) - 25 50 - 25 50
KD
100
VCAM1
50
35
KD
100
RelB
55
35
KD
100
Tubulin
55
35

Fig. 2E

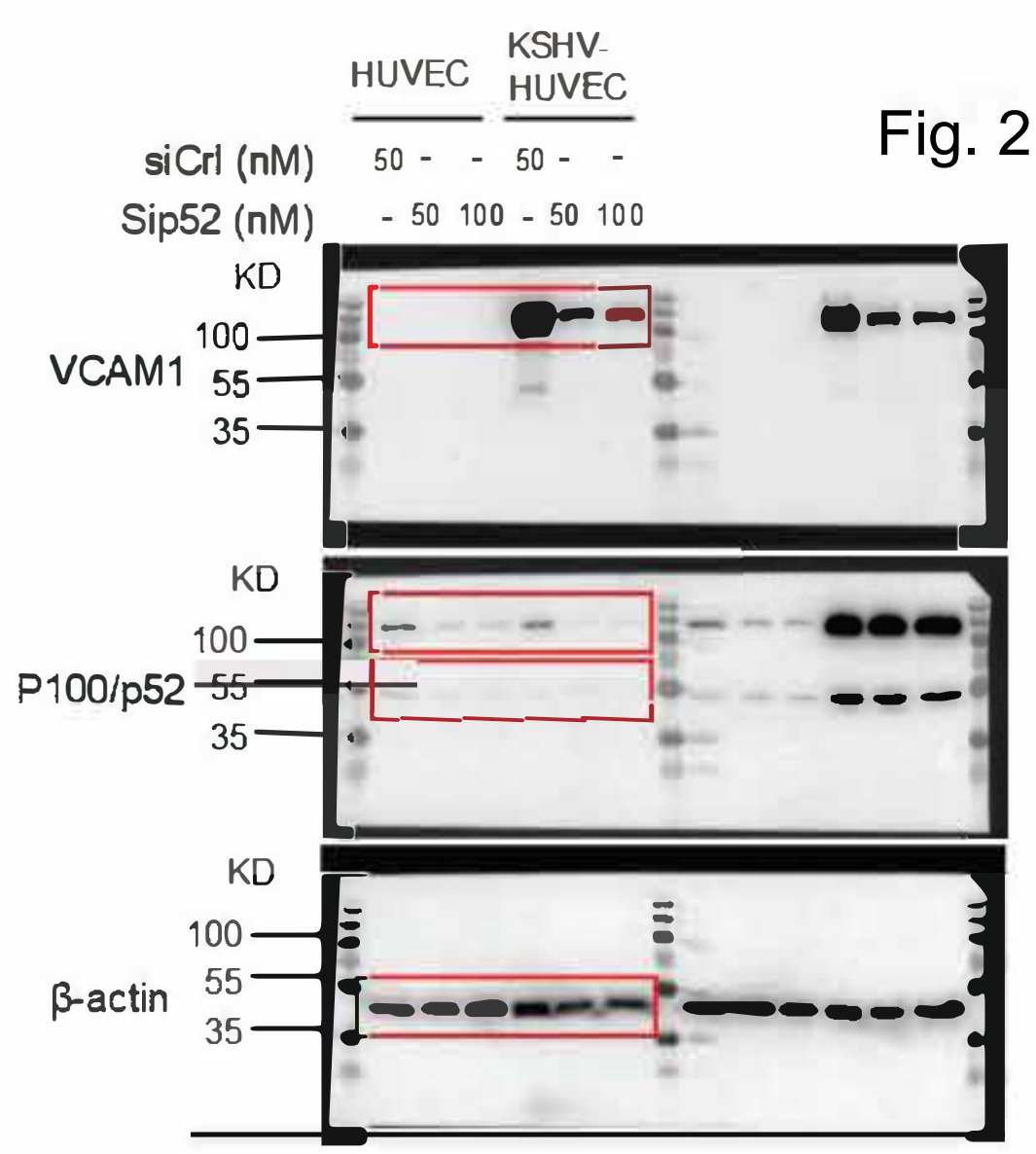
HUVEC
KSHV-HUVEC
siCrl (nM) 50 - - 50 - -
Sip52 (nM) - 50 100 - 50 100
KD
100
VCAM1
55
35
KD
100
P100/p52
55
35
KD
100
β-actin
55
35

Fig. 2F

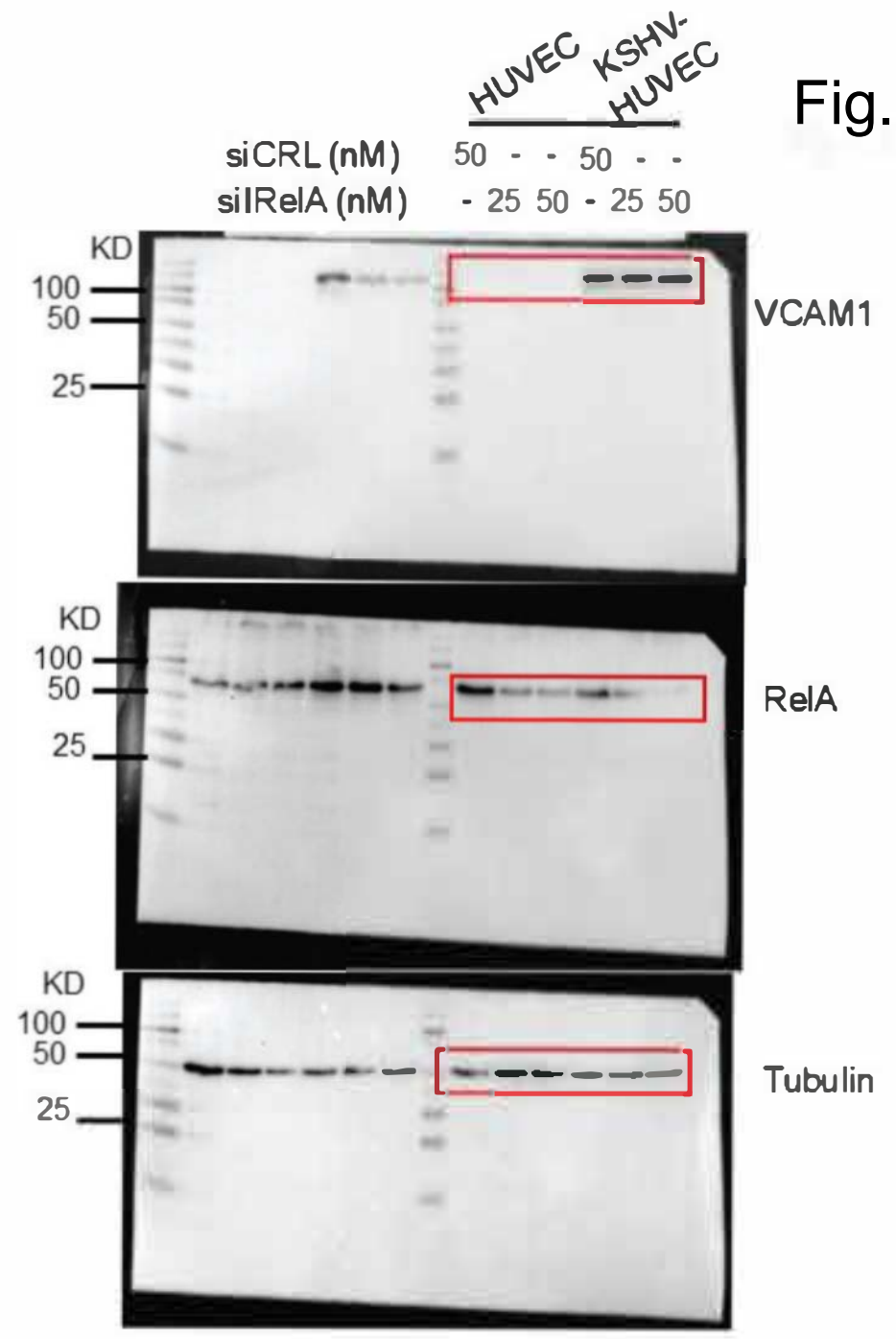

Fig. 2G

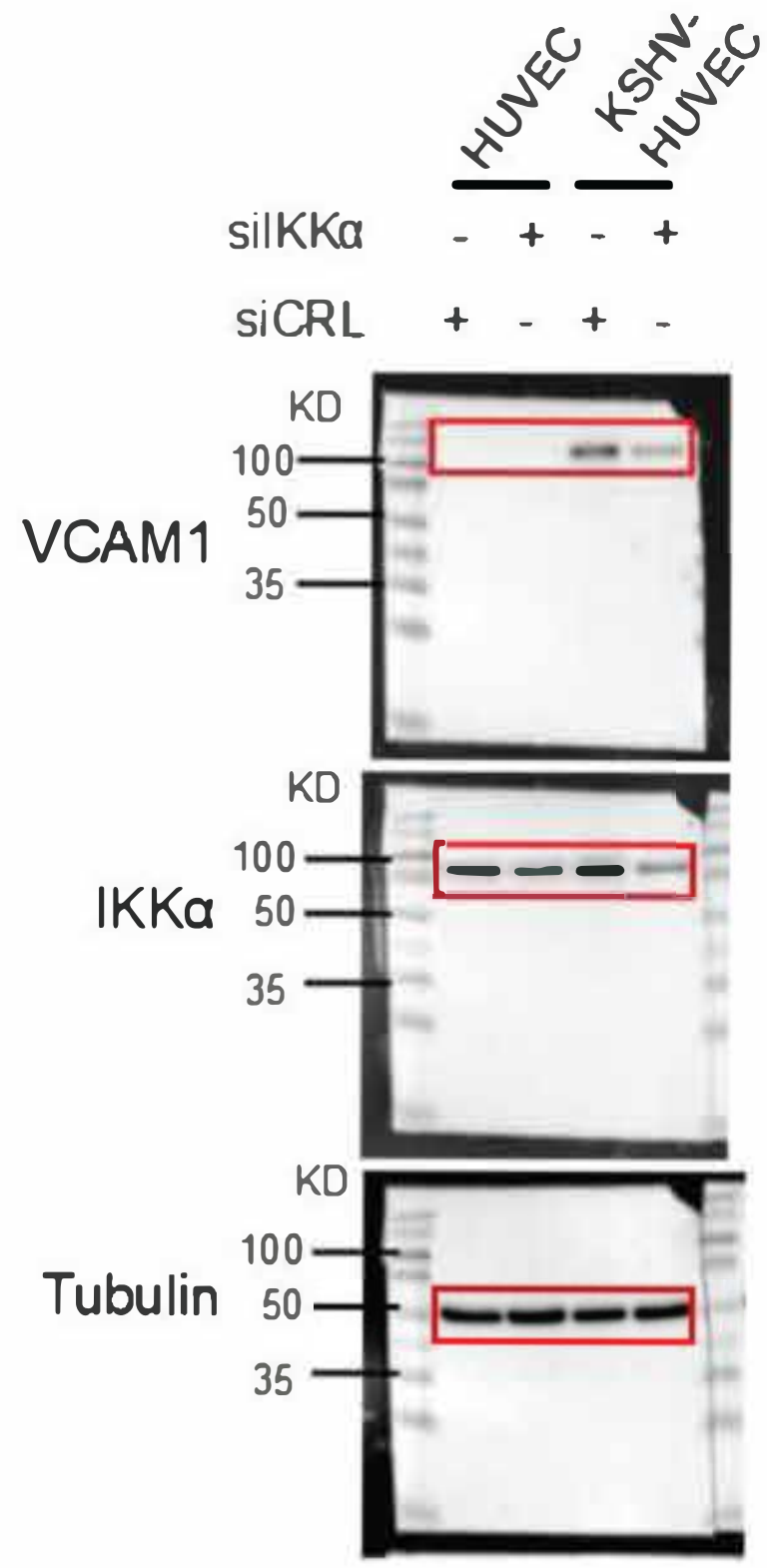

Fig. 2J

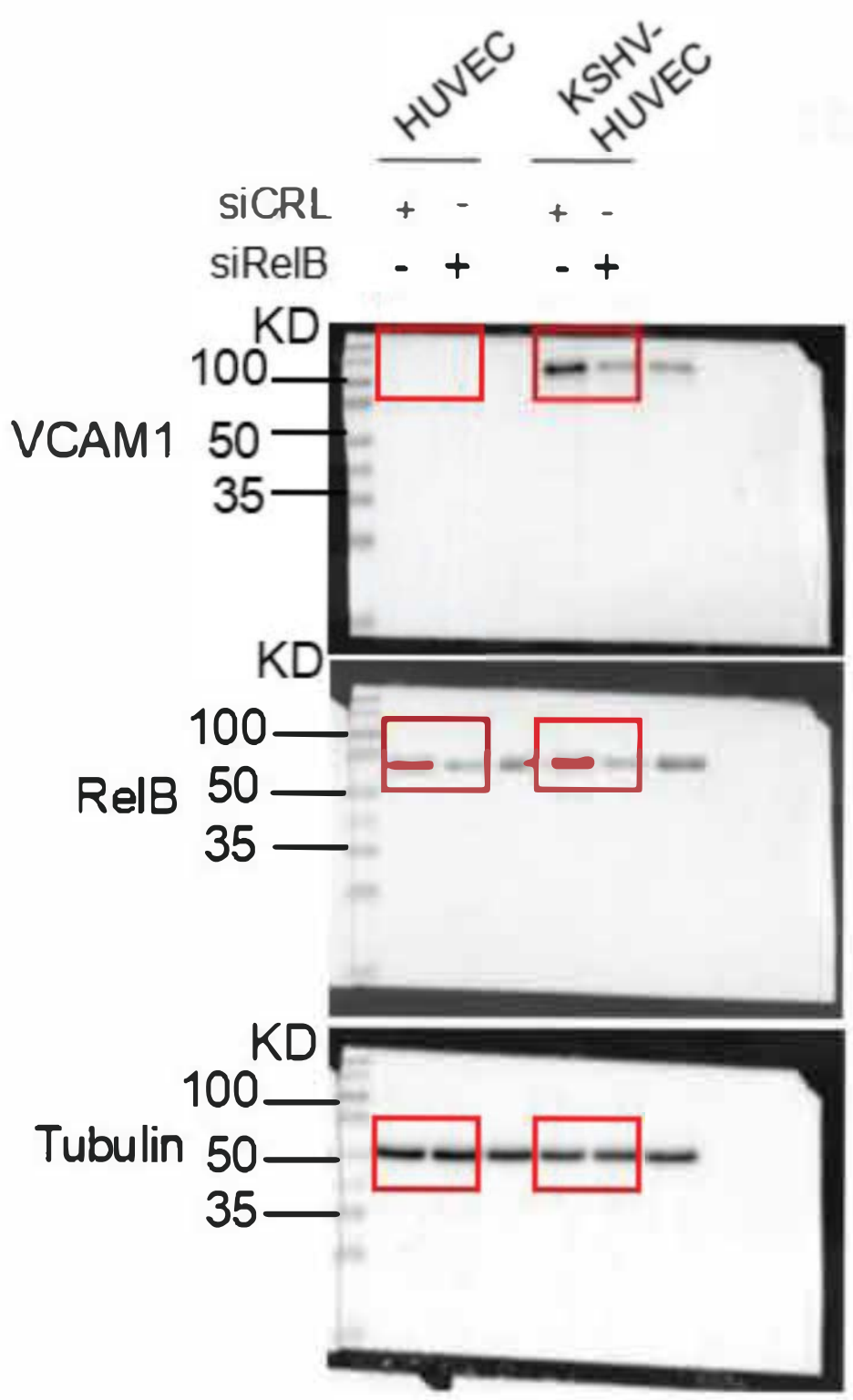

Fig. 2M

Fig. 3C

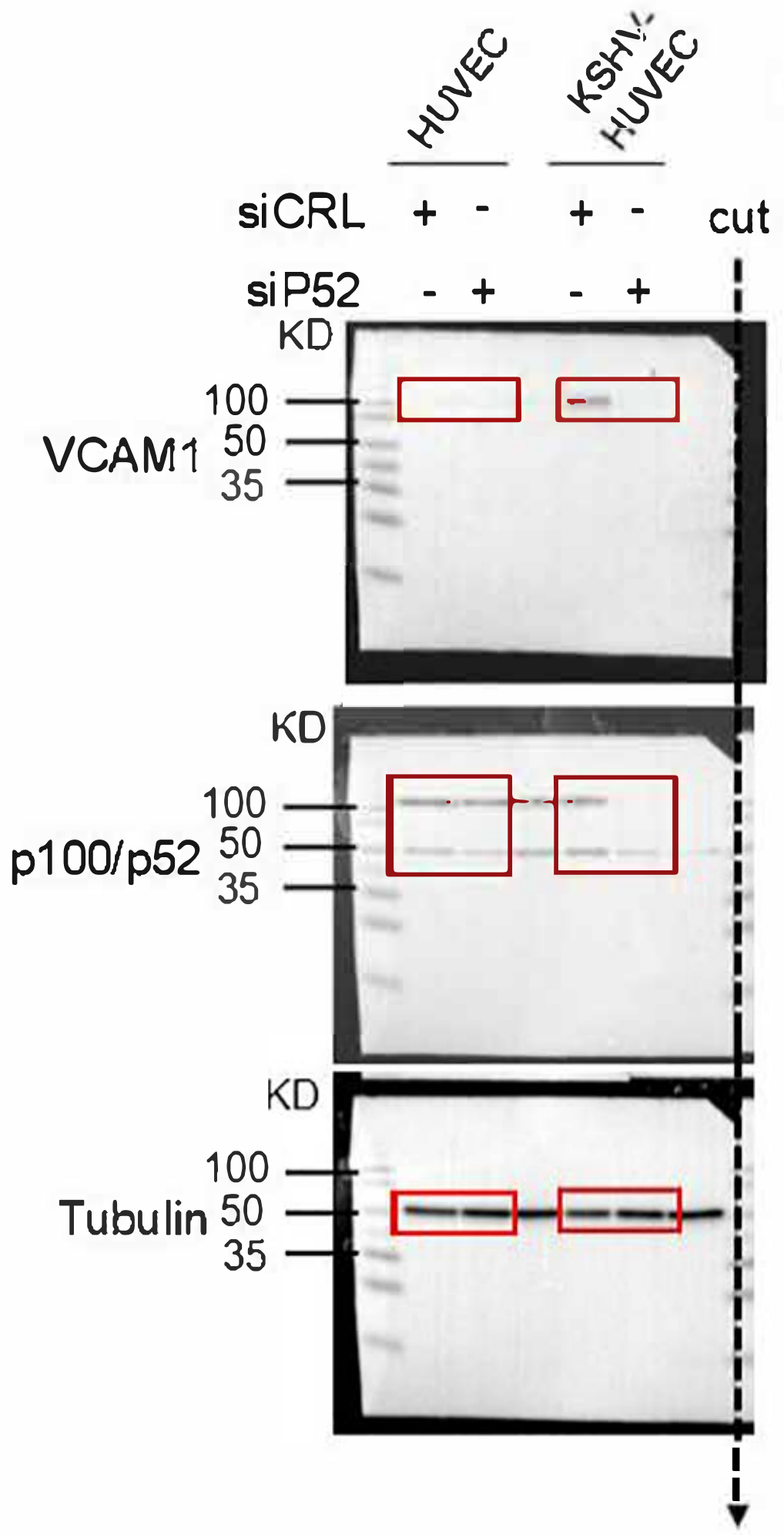

HUVEC
KSHV-HUVEC
siCRL + - + -
siP52 - + - +
cut
KD
100
50
35
VCAM1
KD
100
50
35
p100/p52
KD
100
50
35
Tubulin

Fig. 3F

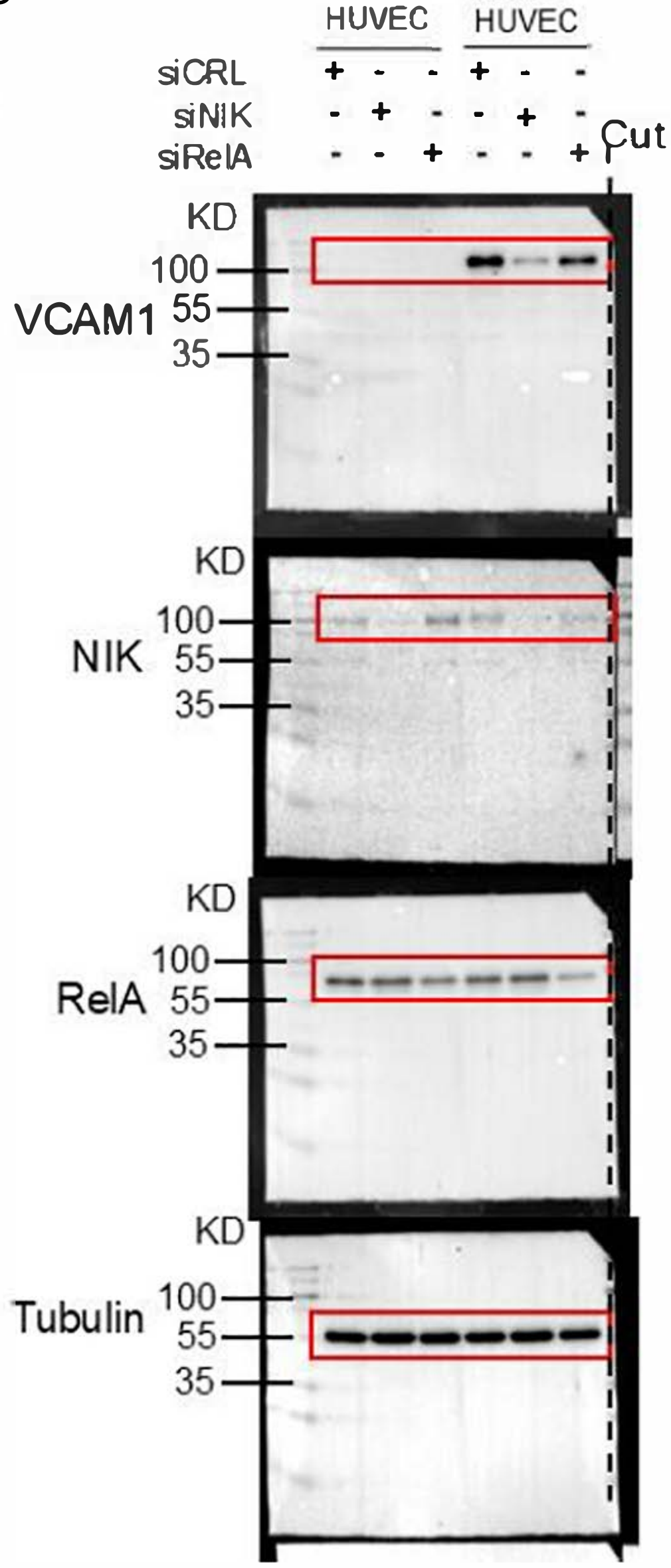

HUVEC
KSHV-HUVEC
siCRL + - - + - -
siNIK - + - - + -
siRelA - - + - - +
Cut
KD
100
55
35
VCAM1
KD
100
55
35
NIK
KD
100
55
35
RelA
KD
100
55
35
Tubulin

Fig. 4C

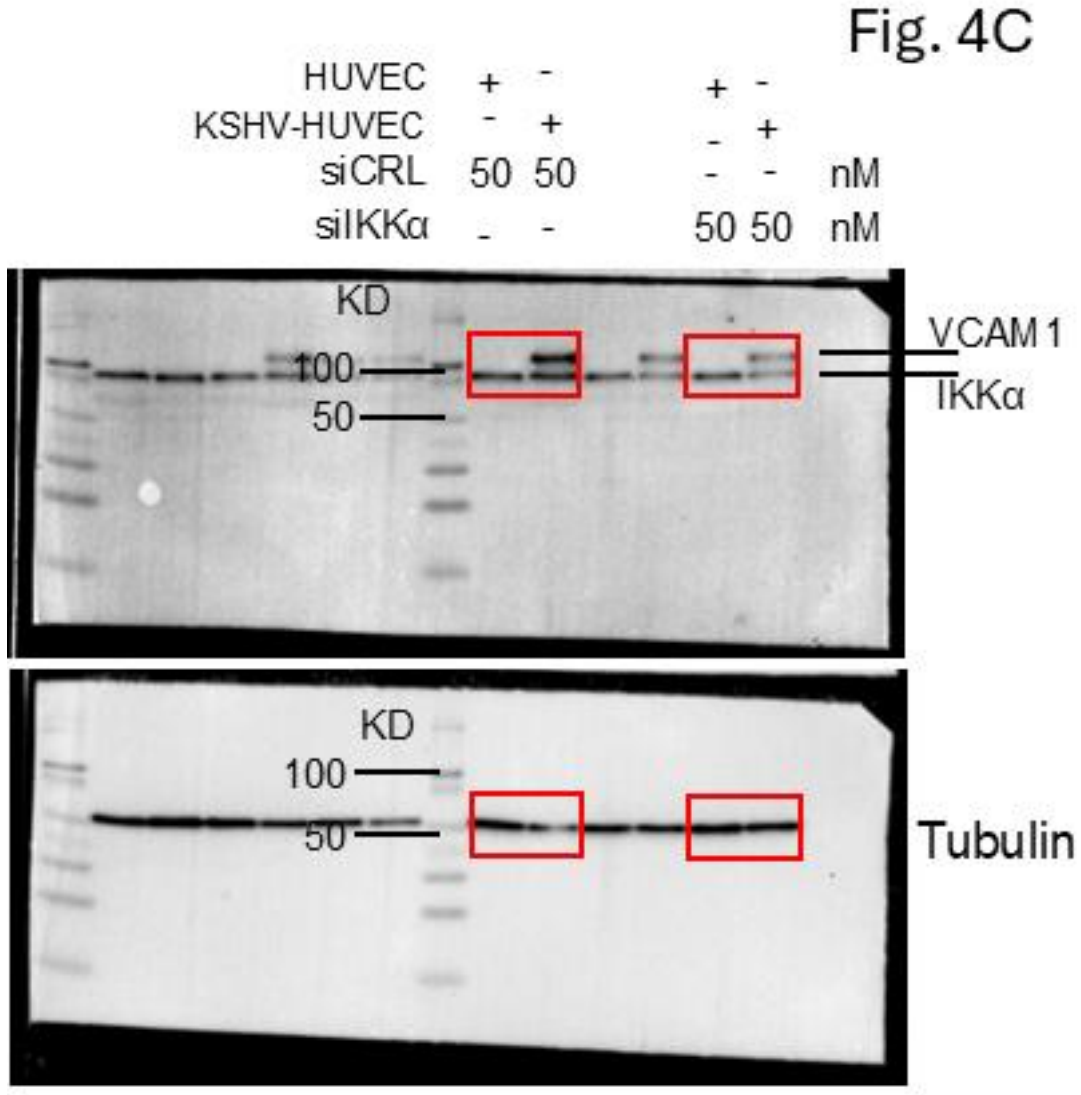

VCAM1 MW = 95-125 KD

IKKα MW = 85-87 KD

Tubulin MW = 55 KD

Protein Markers: cat# 26634

Fig. 5B

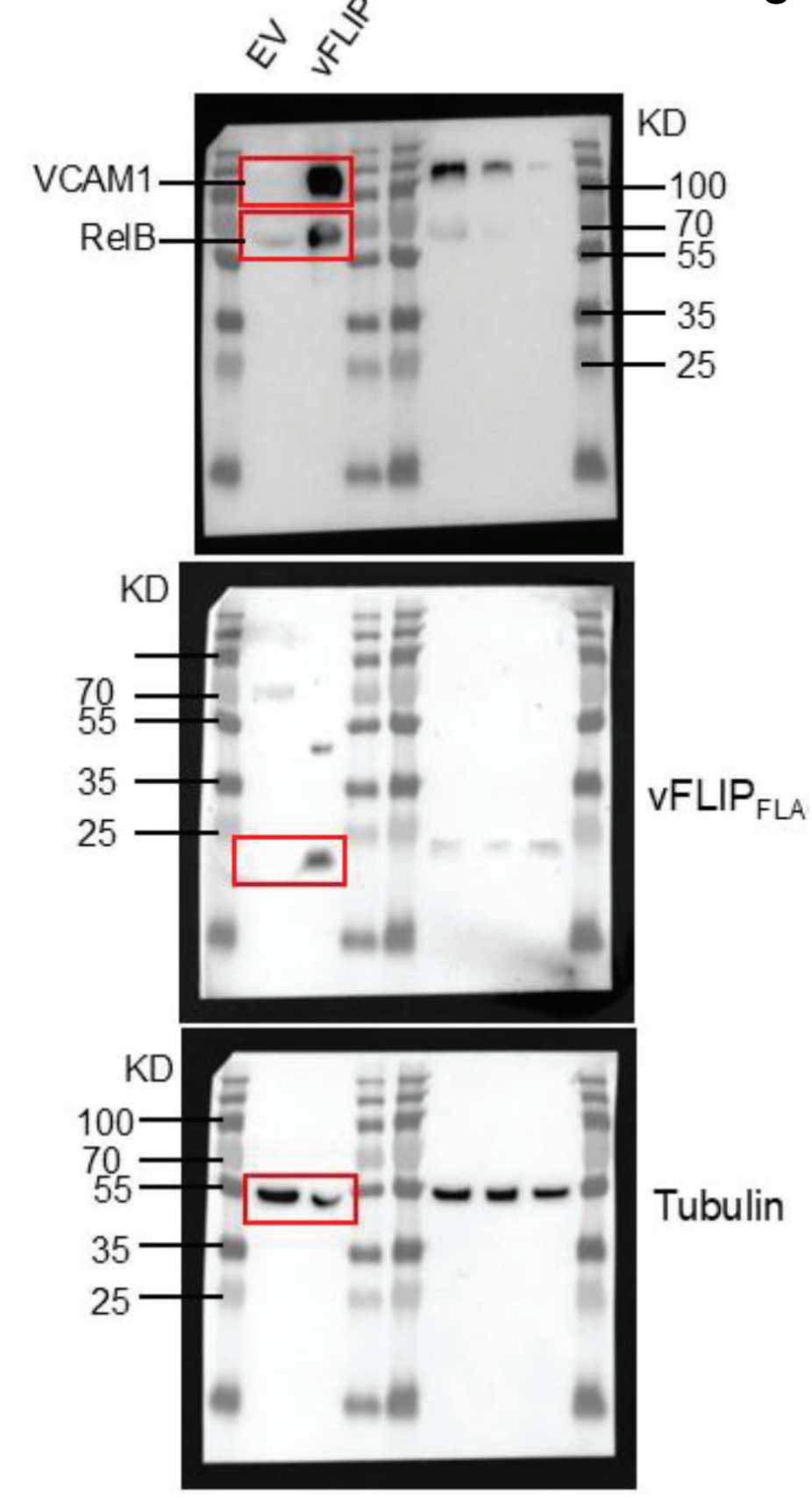
EV
vFLIP
KD
VCAM1
RelB
100
70
55
35
25
KD
70
55
35
25
vFLIPFLA
KD
100
70
55
35
25
Tubulin

Fig. 5E

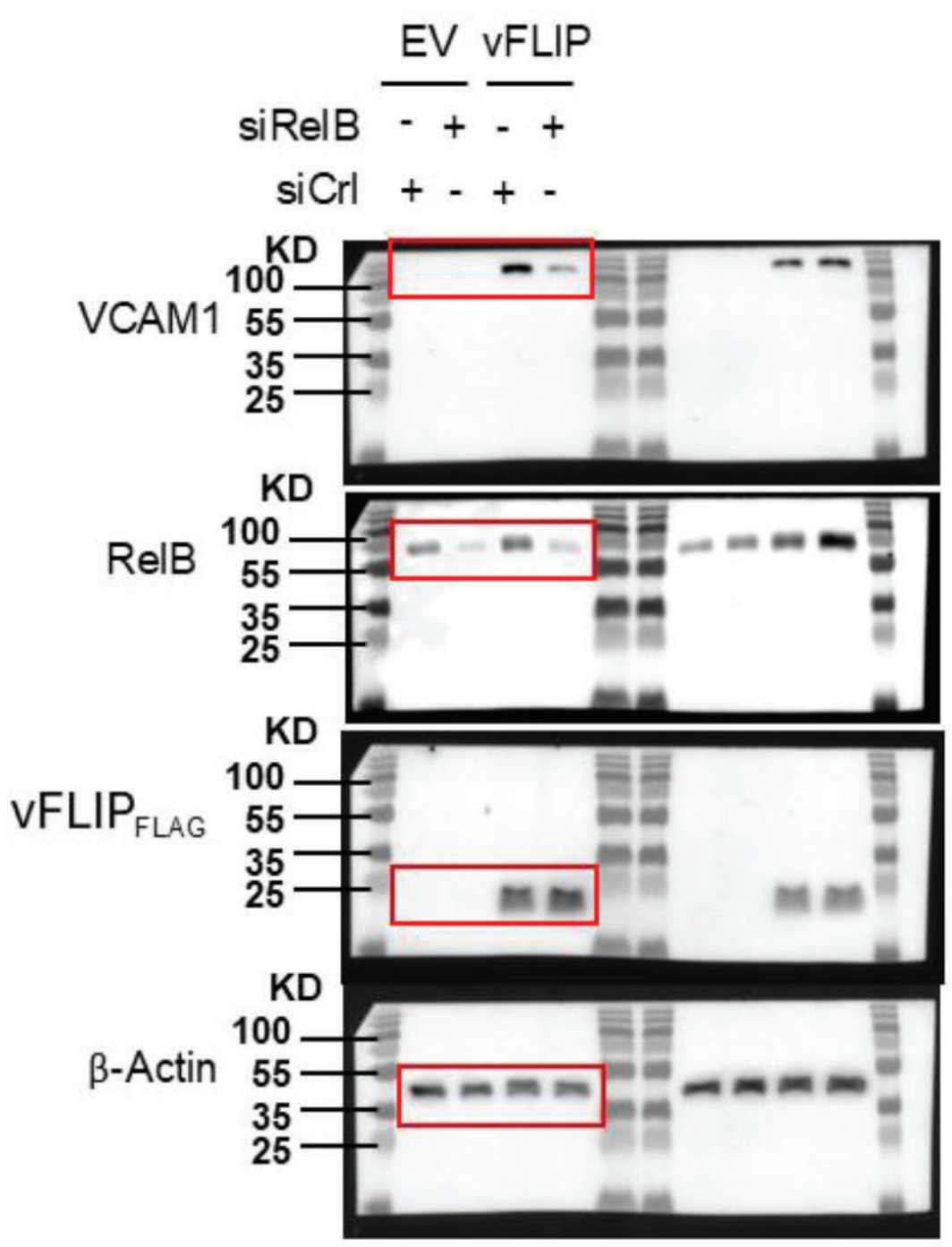
EV
vFLIP
siRelB - + - +
siCrl + - + -
KD
100
VCAM1
55
35
25
KD
100
RelB
55
35
25
KD
100
vFLIPFLAG
55
35
25
KD
100
β-Actin
55
35
25

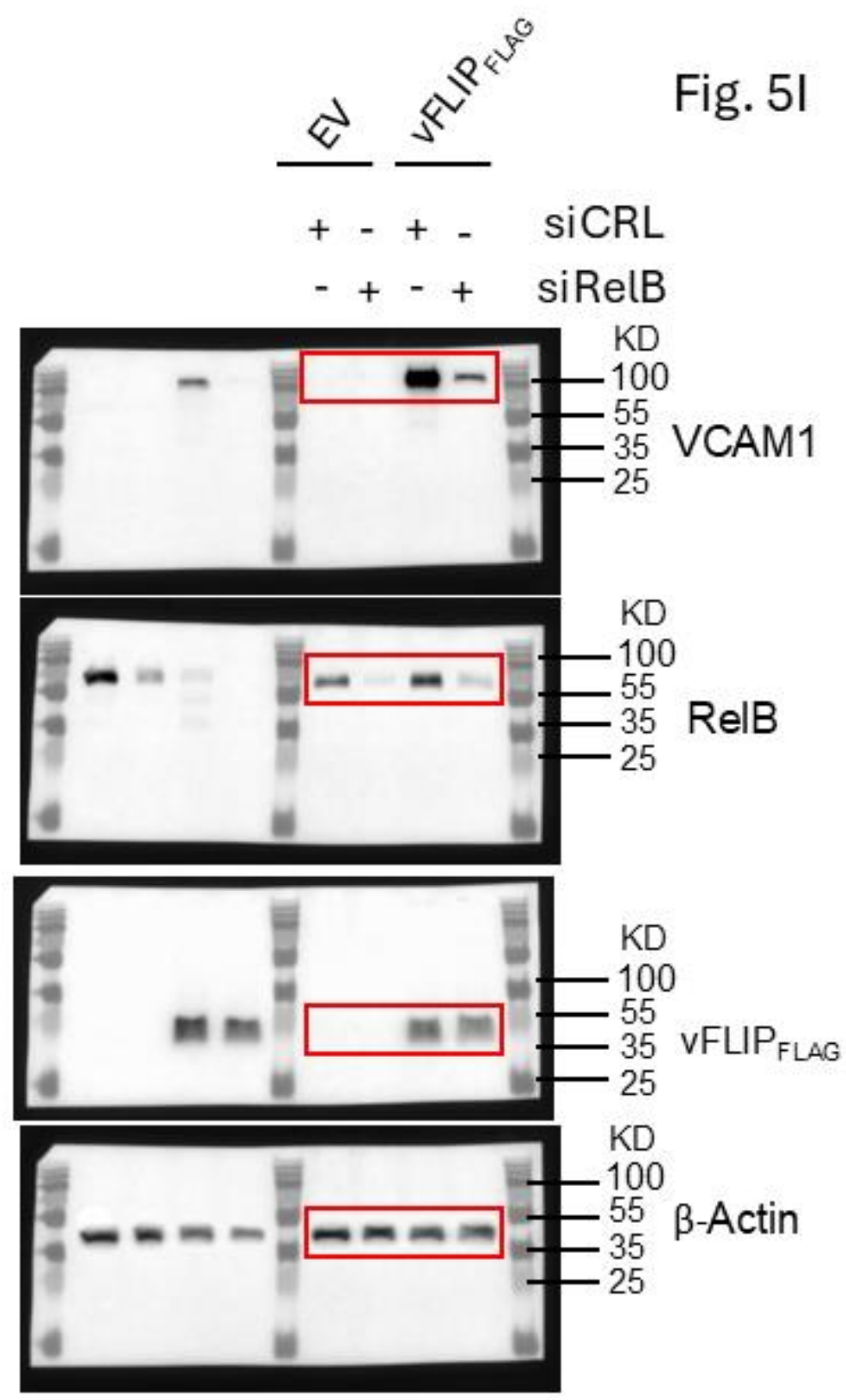

Fig. 5I
EV
vFLIP$_{FLAG}$
siCRL
siRelB
KD
100
55
35
25
VCAM1
RelB
vFLIP$_{FLAG}$
β-Actin

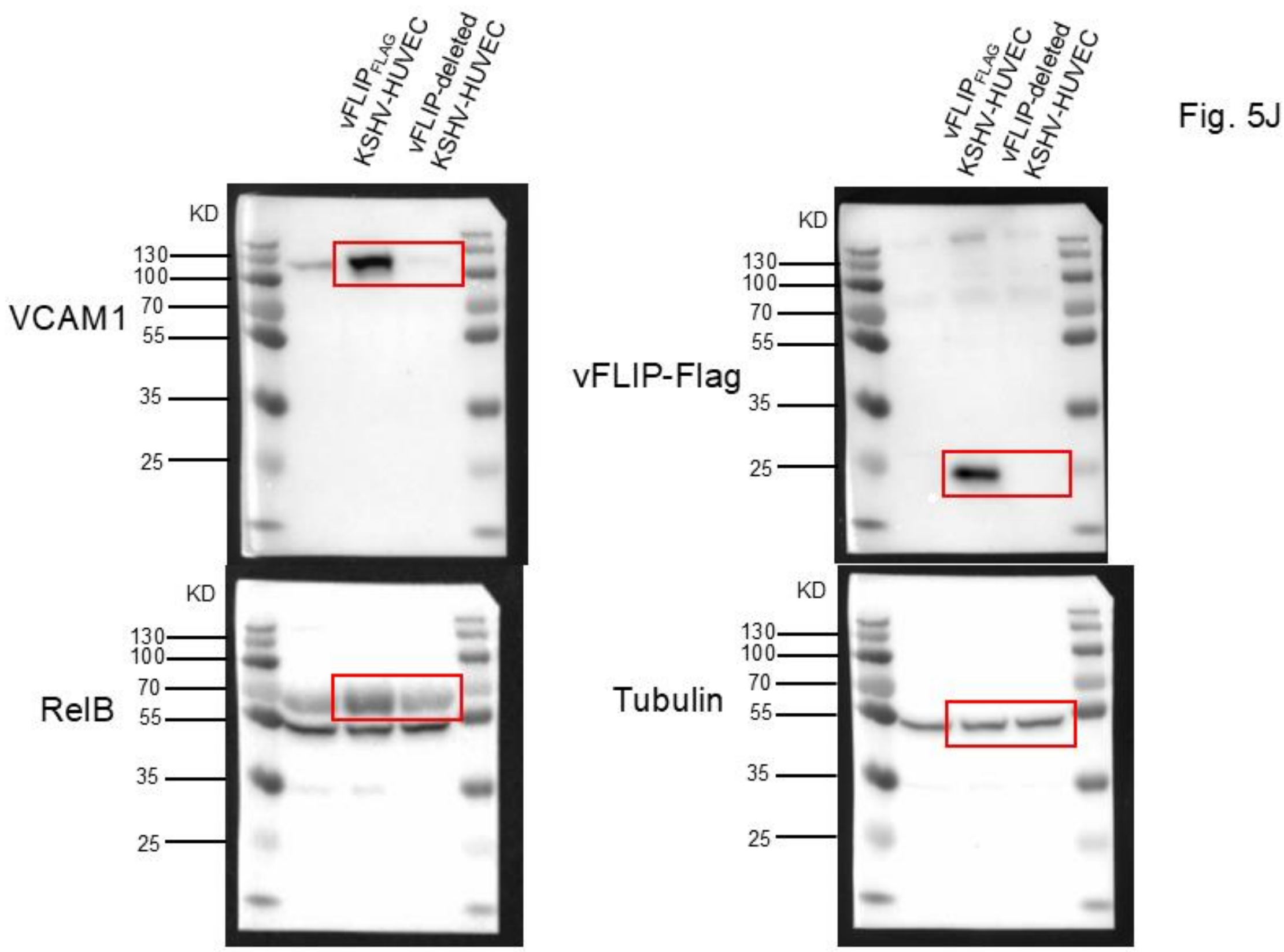

Fig. 5J
vFLIP$_{FLAG}$ KSHV-HUVEC
vFLIP-deleted KSHV-HUVEC
KD
130
100
70
55
35
25
VCAM1
RelB
vFLIP-Flag
Tubulin
